# Ydj1 interaction at nucleotide-binding-domain of yeast Ssa1 impacts Hsp90 collaboration and client maturation

**DOI:** 10.1371/journal.pgen.1010442

**Published:** 2022-11-09

**Authors:** Deepika Gaur, Navinder Kumar, Abhirupa Ghosh, Prashant Singh, Pradeep Kumar, Jyoti Guleria, Satinderdeep Kaur, Nikhil Malik, Sudipto Saha, Thomas Nystrom, Deepak Sharma

**Affiliations:** 1 Council of Scientific and Industrial Research-Institute of Microbial Technology, Chandigarh, India; 2 Institute for Biomedicine, Sahlgrenska Academy, Centre for Ageing and Health-Age Cap, University of Gothenburg, Gothenburg, Sweden; 3 Division of Bioinformatics, Bose Institute, Kolkata, India; 4 Pharmacology Department, School of Science and Technology, Nottingham Trent University, Nottingham, United Kingdom; 5 Department of Biochemistry, School of Interdisciplinary and Applied Life Sciences, Central University of Haryana, Mahendergarh, India; 6 Academy of Scientific & Innovative Research, Ghaziabad, India; Northwestern University, UNITED STATES

## Abstract

Hsp90 constitutes one of the major chaperone machinery in the cell. The Hsp70 assists Hsp90 in its client maturation though the underlying basis of the Hsp70 role remains to be explored. In the present study, using *S*. *cerevisiae* strain expressing Ssa1 as sole Ssa Hsp70, we identified novel mutations in the nucleotide-binding domain of yeast Ssa1 Hsp70 (Ssa1-T175N and Ssa1-D158N) that adversely affect the maturation of Hsp90 clients v-Src and Ste11. The identified Ssa1 amino acids critical for Hsp90 function were also found to be conserved across species such as in *E*.*coli* DnaK and the constitutive Hsp70 isoform (HspA8) in humans. These mutations are distal to the C-terminus of Hsp70, that primarily mediates Hsp90 interaction through the bridge protein Sti1, and proximal to Ydj1 (Hsp40 co-chaperone of Hsp70 family) binding region. Intriguingly, we found that the bridge protein Sti1 is critical for cellular viability in cells expressing Ssa1-T175N (A1-T175N) or Ssa1-D158N (A1-D158N) as sole Ssa Hsp70. The growth defect was specific for *sti1*Δ, as deletion of none of the other Hsp90 co-chaperones showed lethality in A1-T175N or A1-D158N. Mass-spectrometry based whole proteome analysis of A1-T175N cells lacking Sti1 showed an altered abundance of various kinases and transcription factors suggesting compromised Hsp90 activity. Further proteomic analysis showed that pathways involved in signaling, signal transduction, and protein phosphorylation are markedly downregulated in the A1-T175N upon repressing Sti1 expression using doxycycline regulatable promoter. In contrast to Ssa1, the homologous mutations in Ssa4 (Ssa4-T175N/D158N), the stress inducible Hsp70 isoform, supported cell growth even in the absence of Sti1. Overall, our data suggest that Ydj1 competes with Hsp90 for binding to Hsp70, and thus regulates Hsp90 interaction with the nucleotide-binding domain of Hsp70. The study thus provides new insight into the Hsp70-mediated regulation of Hsp90 and broadens our understanding of the intricate complexities of the Hsp70-Hsp90 network.

## Author summary

Hsp70-Hsp90 constitutes major cellular chaperone machinery in cells. The Hsp70 plays critical role in Hsp90 chaperoning pathway. We have now identified novel mutations in the nucleotide-binding domain of yeast Ssa1 Hsp70 (Ssa1-T175N and Ssa1-D158N) that adversely affect Hsp90 client maturation. As compared to wt Ssa1, the identified Ssa1 mutants bind relatively better with Ydj1, and poorly support growth in the absence of Sti1, when present as the sole source of Ssa Hsp70 in *S*. *cerevisiae*. The cells expressing Ssa1-T175N as sole Ssa Hsp70 show downregulation of pathways involved in signaling, signal transduction, and protein phosphorylation upon repressing Sti1. The study shows that Ydj1 interaction at the nucleotide-binding domain of Ssa1 Hsp70 influences Hsp90 function.

## Introduction

The Hsp90 family of chaperones are highly conserved across species, and essential for cellular viability in eukaryotes. About 10% of the yeast proteome is dependent on Hsp90 for its maturation and includes the family of kinases, growth hormone receptors and transcription factors [1]. These clients are involved in a number of cellular processes such as cell cycle control, cellular survival and signalling pathways [2–4]. For many of these substrates, Hsp90 is required for maturation, and for others it is involved in their transport or assembly into multiprotein complex. Hsp90 functions in coordination with another major cellular chaperone Hsp70 which first interacts with client proteins, and remodels them for further maturation by Hsp90s [5]. The dynamics of Hsp70-Hsp90 interactions and its functional significance are under intense investigation.

Each protomer of the homodimeric Hsp90 consists of a highly conserved nucleotide-binding domain (NBD), a middle domain (MD) and a C-terminal domain (CTD). In the absence of ATP, Hsp90 adopts an open V-shape conformation having dimerized CTD with NBDs of each protomer away from each other [6]. The ATP binding at NBD leads to conformational changes that cause the N-terminal domain to transiently dimerize and associate with the middle domain. Also, a segment of NBD folds over the nucleotide-binding pocket and interacts with ATP. The long charged linker consisting of 56 amino acids present between the nucleotide-binding domain and the middle domain of Hsp90 contributes to flexibility towards these conformational changes in the protein [7]. The substrate primarily interacts with MD of Hsp90 [8]. The binding of Hsp90 to its co-chaperone such as Aha1 or Hch1 stimulates ATP hydrolysis which facilitates the release of substrates and thus the initiation of a new reaction cycle [9,10].

Hsp90 is assisted by different co-chaperones that either modulates its ATPase activity or facilitate substrate transfer as well as conformational changes. In *S*. *cerevisiae*, the heterocomplex Hsp70-Sti-Hsp90 is required for substrate transfer from Hsp70 to Hsp90 where Sti1 acts as the bridge protein and interacts simultaneously with MEEVD motif present at the C-terminus of Hsp90 and C-terminal EEVD motif of ADP bound Hsp70 [11,12]. Additionally, Sti1 is also known to act as an inhibitor of Hsp90 basal ATPase activity [13]. As a substrate is transferred to Hsp90, Sti1 dissociates from Hsp90 and is replaced by another TPR domain-containing protein, such as Cpr6, Cpr7 or Cns1, as Hsp90 traverses through different stages. Cns1 is essential for cellular viability, and deletion of Cpr7 results in temperature sensitivity [14]. Another TPR protein Tah1 in complex with Pih1 interacts with Hsp90 and regulates its ATPase activity [15].

Hsp70 interacts with partially unfolded substrates to prevent their aggregation, and promote folding back to the native state. Many of these substrates are further transferred to Hsp90. The additional requirement of Hsp90 action for some Hsp70 substrates is still not clear. Unlike eukaryotes, prokaryotes lack homologs of the bridging protein Sti1 that bridges Hsp70 with Hsp90. Instead, a direct interaction between the *E*.*coli* Hsp70 (DnaK) and Hsp90 (HtpG) has been observed [16]. Similar interaction has also been seen for yeast and mammalian Hsp70 and Hsp90 [17,18]. One study showed that direct interaction between *E*.*coli* Hsp90-Hsp70 results in conformational changes in both proteins [19]. Further, these direct interactions lead to synergistic stimulation of ATP hydrolysis activity and are required for client reactivation in vitro [20]. It has been shown also that cells carrying mutations in the middle domain of the yeast Hsp90 that disrupt its interactions with NBD of Hsp70 are unable to grow at 37°C [17]. Further, in the absence of Sti1, these Hsp90 mutants are unable to support growth even at the optimal temperature of 30°C [17]. However, the molecular basis of this importance of Hsp70-Hsp90 interactions remains unclear.

Similar to Hsp90, the Hsp70 family of proteins are highly conserved across different species; from prokaryotes to mammals. Hsp70 chaperones are present in different cellular compartments including the cytosol, endoplasmic reticulum, lysosomes, and mitochondria, and perform a variety of functions including those linked to protein trafficking, cellular signaling, protein folding and degradation [21]. Hsp70 consists of three domains; the N-terminal nucleotide-binding domain, the substrate-binding domain and a C-terminal domain that acts as a lid over the substrate-binding domain. Similar to Hsp90, the Hsp70 reaction cycle is regulated by its interaction with various co-chaperones. The co-chaperones not only regulate Hsp70 activity but also provide functional specificity to different Hsp70 isoforms. Hsp40 assists in substrate transfer as well as stimulates Hsp70 ATPase activity [22,23]. Nucleotide exchange factors, in turn, exchange ADP with ATP which facilitates substrate release from Hsp70 to initiate a new reaction cycle [24,25].

In the present study, we attempted to elucidate the requirement of different structural regions of Hsp70 in Hsp90 chaperoning activity. To examine Hsp70 role, we employed *S*. *cerevisiae* strain expressing Ssa1, and lacking remaining three isoforms, as sole source of Ssa Hsp70. Using random mutagenesis, we isolated various Hsp70 mutants defective in their role in the maturation of the Hsp90 client protein v-Src. We show that the Hsp70 mutants were defective in their direct interaction with Hsp90, and have enhanced interaction with Ydj1. The data suggest that Hsp90 direct interaction at nucleotide-binding domain of Hsp70 is regulated by Hsp40 binding at the same region of Hsp70. The Ydj1-mediated regulation of Hsp70-Hsp90 interaction plays a crucial role in client protein maturation and thus cell survival under stress conditions.

## Results

### Identification of Ssa1 residues critical for the maturation of Hsp90 clients

Hsp70 plays a critical role in the maturation of Hsp90 clients such as v-Src, Ste11 and many growth hormone receptors [26–28]. *S*.*cerevisiae* encodes for four highly homologous members of cytosolic Ssa Hsp70 members. To examine the Hsp70 region critical for Hsp90 function, we used the yeast strain A1 that lacks all four chromosomally encoded Ssa1-4 Hsp70s and expresses Ssa1 from a plasmid-encoded gene, as the sole Ssa Hsp70 source. The Rous sarcoma viral tyrosine kinase, v-Src was used as an Hsp90 client. The heterologous overexpression of matured v-Src leads to growth arrest in *S*.*cerevisiae* due to uncontrolled phosphorylation of tyrosine residues present in most of the cellular proteins [29]. Similar to a previous report, overexpression of v-Src in the A1 strain from a galactose-inducible promoter led to poor growth onto solid SGal media (S1A Fig) [26]. To identify Ssa1 residues required for Hsp90 activity, we randomly mutagenized Ssa1 to isolate mutants that suppress v-Src-mediated toxicity. The plasmid-encoding Ssa1 was mutagenized and the mutant library was transformed into a strain harbouring galactose inducible v-Src and expressing wt Ssa1 from the *tet* repressible promoter. The transformants were plated onto doxycycline plate to repress wt Ssa1 expression and colonies that showed optimal growth onto solid SGal media were selected for further analysis (S1A Fig). The plasmids were extracted from well-grown colonies, and reconfirmed for their ability to suppress v-Src-mediated toxicity.

**Fig 1:**
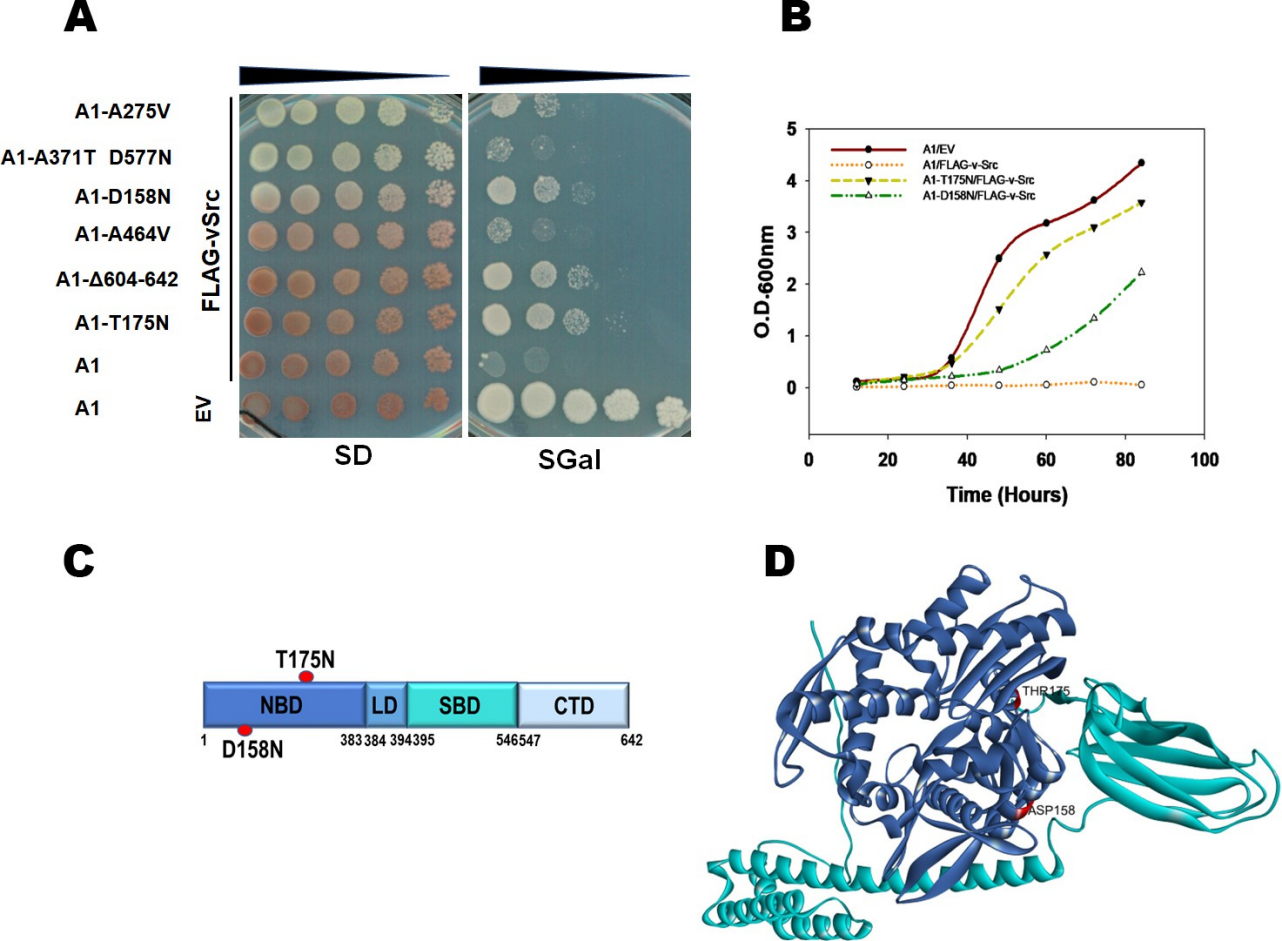
Strains expressing Ssa1-T175N or Ssa1-D158N show reduced v-Src toxicity. **(A)** *S*. *cerevisiae* strains expressing indicated Ssa1 allele as the sole source of Ssa Hsp70. The v-Src expression was induced using galactose (SGal). **(B)** Cell growth in SGal media at 30°C. Shown is O.D._600nm_ at various time intervals. **(C)** Schematics showing domain architecture and position of identified mutations (D158N and T175N) in Ssa1. **(D)** The 3D structure of Ssa1 from AlphaFold database where NBD is coloured blue and amino acids at 158 and 175 are marked in red.

Six Ssa1 alleles displayed a reproducible reduction of v-Src toxicity (Fig 1A and Table 1). We further chose to focus on particular Ssa1 alleles on the basis that i) the selected mutant showed relatively strong suppression of v-Src toxicity, and ii) the mutation was located at a site distal to that known to interact with the bridging protein Sti1. The Hsp70 mutations distal to the Sti1 interaction site was chosen not to disrupt the Hsp70-Hsp90 interaction mediated by the bridge protein. Among the 6 isolated mutants, A1-Δ604–642 showed insertion of a stop codon at the 604^th^ residue leading to deletion of 39 residues at the C-terminal end of Ssa1. This mutant lacks the EEVD motif present at the C-terminus of Hsp70 required for interaction with Sti1. Among the remaining mutants, A1-T175N and A1-D158N showed reproducibly better suppression of v-Src toxicity and thus used for further studies (Figs 1A and S2). Both T175 and D158 residues were found to be conserved among different Ssa Hsp70 isoforms (Ssa1-4 and human Hsc70 and Hsp70 (S1B Fig).

**Table 1:** Amino acid homologous to Ssa1 mutants, in Human HspA8 and *E*.*coli* DnaK, showing suppression of v-Src mediated toxicity.

Ssa1 mutants	DnaK	HspA8
A1-T175N	T173	T177
A1-Δ605–642	-	-
A1-A464V	A465	A467
A1-D158N	D156	D160
A1-A371T D577N	A376, Not conserved	A374, D582
A1-A275V	Not conserved	Not conserved

The ability of A1-T175N and A1-D158N to reduce the v-Src-mediated growth defect was further confirmed by the growth of the strains expressing either of the two mutant proteins as the sole source of Ssa Hsp70 and overexpressing v-Src. As shown in Fig 1B (S1 Dataset), the A1 strain overexpressing v-Src grew poorly whereas those expressing the T175N (A1-T175N) or D158N (A1-D158N) mutant Ssa1s grew markedly better. As seen, the presence of Ssa1-T175N had a more pronounced effect than Ssa1-D158N in reducing v-Src toxicity.

We further examined whether the effect of Ssa1-T175N and Ssa1-D158N on v-Src toxicity was dominant or recessive. The plasmid encoding wt Ssa1 or the mutant alleles were transformed into a wt strain harboring plasmid encoding galactose inducible FLAG-v-Src and all four chromosomally-encoded Ssa Hsp70s. The transformants were grown in liquid SD media, and then serially diluted onto solid media with dextrose or galactose as the carbon source. As seen in S3 Fig, as compared to wt cells lacking v-Src, significant growth defect was observed in cells upon v-Src expression. The expression of either wt Ssa1 or its mutant alleles could not rescue cells from v-Src-mediated toxicity. These results suggest that the effect of Ssa1-T175N and Ssa1-D158N on v-Src mediated growth defect is recessive.

The experimental 3D structure of Ssa1 is not yet known, we have used a modelled structure available in AlphaFold database (https://alphafold.ebi.ac.uk/entry/P10591). Fig 1D shows the Ssa1 structure with the location of the D158N and T175N mutations. Interestingly, both the mutations lie in the nucleotide-binding domain, and not the substrate-binding domain of Ssa1 (Fig 1C).

### v-Src maturation is reduced in A1-T175 and A1-D158

**Fig 2:**
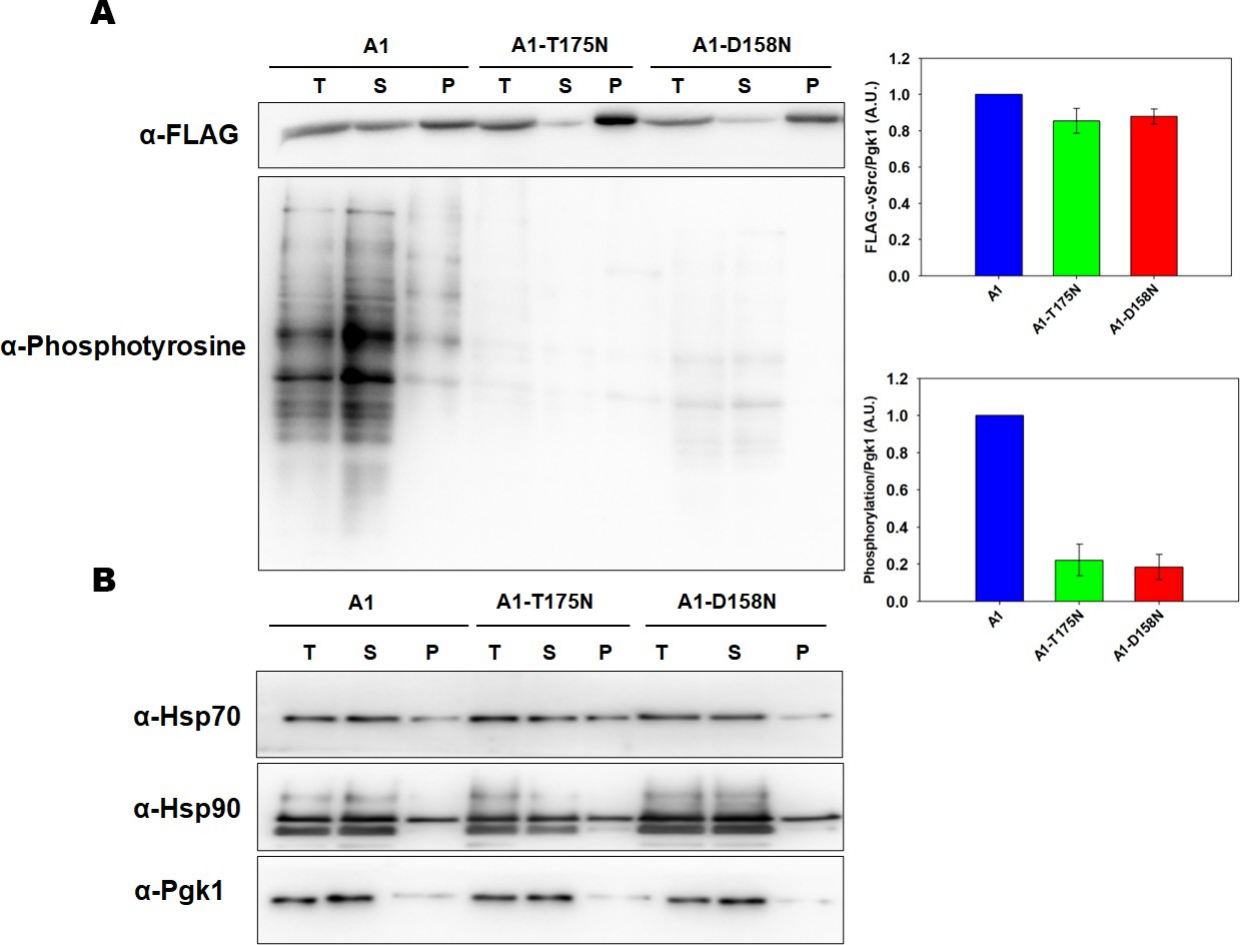
A1-T175N and A1-D158N show reduced v-Src kinase activity. **(A)** Indicated strains were grown in SGal liquid media for 6h. The whole cell lysate (T) was prepared, and fractionated into supernatant (S) and Pellet (P). All fractions were probed with anti-FLAG or anti-phosphotyrosine antibody. Panel towards right depicts quantification from immunoblots. **(B)** The Hsp70, Hsp90 or Pgk1 was probed with anti-Hsp70, anti-Hsp90 or anti-Pgk1 antibody respectively. Pgk1 is the loading control and is same for 2A and 2B. Error bars represent standard error from 3 different biological replicates.

The above results show that v-Src-mediated toxicity is reduced in strains expressing Ssa1-T175N or Ssa1-D158N. To examine whether the reduced toxicity is due to poorer maturation of v-Src, we overexpressed v-Src in strains expressing wt or mutant Ssa1, and measured the kinase abundance and activity using anti-FLAG and anti-phosphotyrosine antibodies, respectively. As shown in Fig 2A (S2 Dataset), v-Src levels were found to be similar in A1, A1-T175 or A1-D158, suggesting that the v-Src abundance was not affected. To elucidate this further, we examined the fraction of v-Src present in the soluble versus the pelleted fraction of the cellular lysate. As seen in Fig 2A (S2 Dataset), though about similar amount of v-Src was present in soluble and pellet fraction in A1 strain, most of the kinase was found as insoluble fraction A1-T175N (~90%) and A1-D158N (~70%) cells suggesting that most of the kinase present in strains with mutant Ssa1 accumulate as inactive aggregates. We further monitored the v-Src kinase activity and found that as compared to A1, the activity was compromised in A1-D158N, and markedly reduced in A1-T175N (Fig 2A) (S2 Dataset).

Previous studies show that inactive v-Src is degraded relatively faster, and thus reduced maturation of v-Src results in lower steady-state levels of the protein [30]. However, our results show that though the kinase activity varies in A1, A1-T175N and A1-D158N, but overall v-Src level in similar in different strains. We further measured the abundance of Hsp70 and Hsp90 which are known to affect v-Src maturation (Fig 2B) (S2 Dataset). As seen, both Hsp70s and Hsp90s are present at similar levels in A1 versus the A1-T175N and A1-D158N strains, suggesting that v-Src aggregation in these strains is unrelated to the changes in expression level of these chaperones.

The diminished maturation of v-Src in the A1-T175N and A1-D158N strains could be due to an effect of the mutation on the Hsp70 structure compromising its ability to promote substrate folding. We thus monitored the secondary structure of Ssa1, Ssa1-T175N and Ssa1-D158N using far-ultraviolet circular dichroism spectroscopy (S5 Fig) (S15 Dataset). The secondary CD is widely used to estimate secondary structural contents such as α-helices and β-sheets in proteins. As seen, the secondary structure of the Ssa1 mutants was found to be similar to that of the wt Ssa1, suggesting that the mutations did not cause any significant alteration to the protein structure.

As heat shock proteins affect v-Src maturation, we examined the abundance of major heat shock proteins such as Ydj1, Hsp104 and Sse1 in cells expressing either of the Ssa1 mutants as the sole Ssa Hsp70 source (S4 Fig). As seen above, the Ssa Hsp70 mutants were found to be expressed at a level similar to wt Ssa1. Similarly, we did not observe any difference in the expression level of other chaperones in A1-T175N or A1-D158N strains when compared to A1.

### Ssa1 mutants impair Ste11 pathway activity

We next examine whether the effect of the Ssa1 mutations (A1-T175N and A1-D158N) on Hsp90 client protein is specific for v-Src or more general, affecting other known Hsp90 client proteins. Ste11, a mitogen-activated protein kinase kinase kinase is a well-known Hsp90 client protein [31]. The reduced maturation of Ste11 results in reduced activation of the transcription factor Ste12. As Ste12 binds to pheromone response elements, a defect in Ste11 maturation adversely affects transcriptional activation of a pheromone-responsive reporter gene. Thus the reporter *lacZ* under control of a promoter containing three repeats of the pheromone response element (PRE-*lacZ*) is widely used to monitor Ste11 maturation [32]. Ste11 contains three N-terminal regulatory domains. A constitutively active Ste11ΔN that lacks these regulatory domains causes inhibition of cell growth due to a combined activation and suppression of mating pathway signalling, and a high osmolarity response signalling, respectively [33].

To examine the effect of Ste11ΔN on the growth of cells expressing different Ssa1 allele, the plasmid expressing the constitutively active kinase under control of galactose-inducible promoter was transformed into yeast strains, and a pool of 10–11 transformants was serially diluted onto solid media containing dextrose or inducer galactose as carbon sources. As shown in S6A Fig, cells expressing Ste11ΔN displayed a growth defect in cells carrying wt Ssa1 (S6A Fig). In contrast, strains expressing either Ssa1-T175N or Ssa1-D158N did not show any significant growth defect, and grew quite similar to the A1 strain lacking the kinase expression (S6A Fig), suggesting reduced maturation of Ste11ΔN in strains expressing the mutant Ssa1s. To examine whether reduced Ste11ΔN-mediated toxicity is related to the kinase expression, we examined the steady state level of Ste11ΔN in A1-T175N and A1-D158N. As evident in S6B Fig, the expression level of His_6_-Ste11ΔN in A1-T175N and A1-D158N is similar to that in A1.

Hsp90 is required for the Ste11 pathway activity [31]. We further examined Ste11ΔN maturation by monitoring its effect on the activity of the reporter enzyme β-galactosidase expressed from the promoter containing PRE sequence. As shown in S6C Fig (S16 Dataset), the strain expressing wt Ssa1 showed significant β-galactosidase activity. As compared to A1, A1-T175 and A-D158 displayed a more than 8 fold reduction in the activity of β-galactosidase suggesting reduced maturation of Ste11ΔN or other client protein in the pathway, in strains expressing the mutant Ssa1 Hsp70s.

We further compared the Ste11 pathway activity in the A1-T175N and A1-D158N strains with that in A1. The A1-T175N, A1-D158N and A1 strains were transformed with a plasmid encoding PRE-*lacZ*, and β-galactosidase activity was monitored as described in Materials and Methods. The A1 strain expressing wt Ssa1 showed a significant increase in β-galactosidase activity in response to α-factor (S6D Fig) (S17 Dataset). As seen, the activity was reduced by more than 40 fold in the strain expressing Ssa1-T175N instead of Ssa1. A similar reduction in pheromone-dependent signalling was observed in A1-D158N, suggesting a poor maturation of Ste11 (or other cellular factor in the pathway) in strains expressing A1-T175N or A1-D158N mutant as the sole Ssa Hsp70 [34].

### v-Src shows enhanced interaction with Hsp70s carrying T175N or D158N mutation

**Fig 3:**
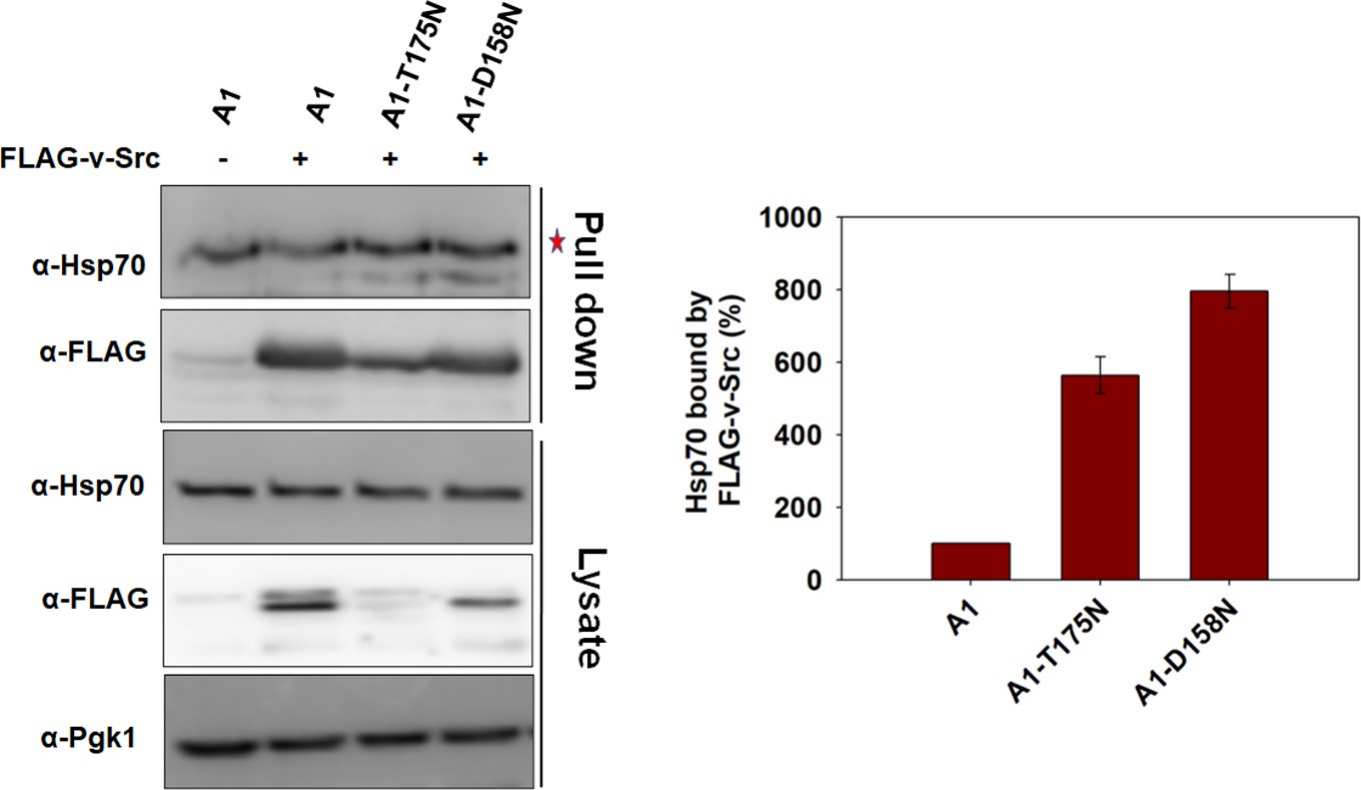
v-Src interaction with Ssa1-T175N and Ssa1-D158N is stronger than with Ssa1. The indicated strains were grown in selective SGal media for v-Src expression. The cellular lysate was incubated with anti-FLAG antibodies immobilized beads. The immunoprecipitated proteins were probed with indicated antibodies. The supernatant was used as a control to examine protein level in A1, A1-T175N and A1-D158N strains. Panel towards right depicts quantification of respective western blots. Star represents nonspecific band. Error bars represent standard error from 3 different biological replicates.

As v-Src is an Hsp90 client, its reduced maturation could be due to an effect on its interaction with Hsp90 in cells expressing mutant Ssa Hsp70 isoforms. We thus monitored the interaction of the kinase client with Hsp90 or Hsp70 by immunoprecipitating FLAG-v-Src using anti FLAG antibody-bound beads. The beads were washed, and bound proteins were probed with antibodies against Hsp70s or Hsp90s. Due to low levels of v-Src in the cell supernatant, we could not capture v-Src-Hsp90 interactions in strains expressing mutant Ssa Hsp70s. However, we could successfully compare client protein interactions with Ssa Hsp70s. As seen in Fig 3 (S3 Dataset), wt Ssa1 interacted with v-Src and such v-Src-Hsp70 interaction increased with the Ssa1-T175N or Ssa1-D158N protein, suggesting that v-Src affinity with the mutant Ssa Hsp70s is relatively higher than with the wt Hsp70.

### T175N or D158N mutation in Ssa1 increases its binding to Ydj1

**Fig 4:**
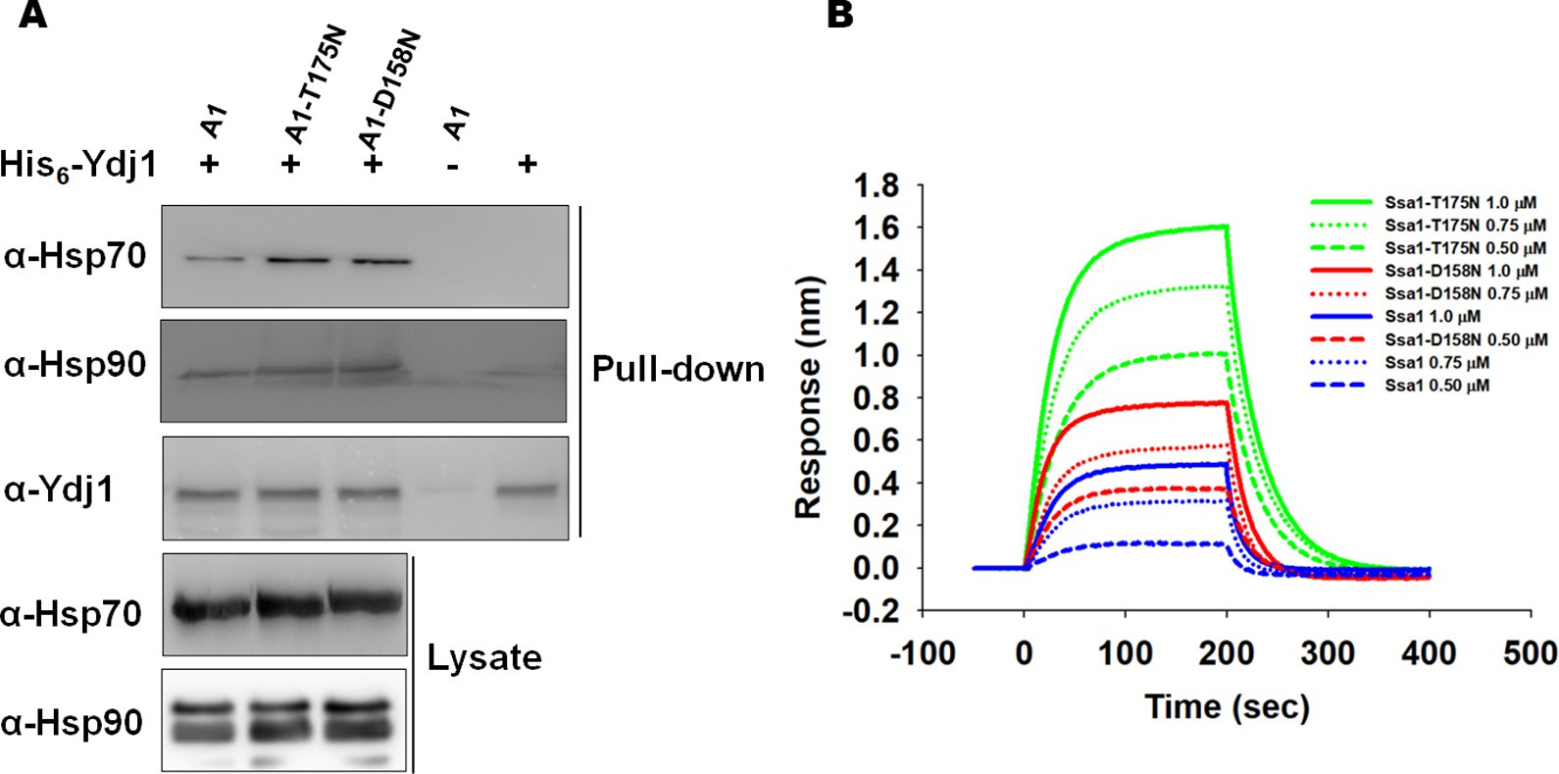
Hsp70 mutants show increased affinity to Ydj1. **(A)** Purified His_6_-Ydj1 was immobilized over Co^2+^-NTA beads and incubated with cellular lysate from indicated strains. The bound proteins were eluted and probed with anti-Hsp70, anti-Hsp90 and anti-Ydj1 antibodies. **(B)** BLI studies to monitor interaction between Ssa Hsp70 and Ydj1. Ydj1 (0.75μM) was immobilized over the biosensor surface, and Ssa Hsp70 (wt Ssa1 or its mutants) was used as analyte. The binding was monitored in the presence of ATP.

Above studies show that v-Src interaction with mutant Ssa Hsp70 is stronger than with wt Ssa1. As the co-chaperone Ydj1 plays a crucial role in v-Src transfer to Ssa Hsp70 [35], we explored whether Ydj1-Hsp70 interaction varied with the mutant Ssa Hsp70s. Purified His_6_-Ydj1 was bound over the Co^2+^-NTA beads, and incubated with cellular lysate from strain expressing wt or mutant Ssa1s. The proteins bound to Ydj1-immobilized beads were eluted and probed on immunoblots with an anti-Hsp70 antibody. As shown in Fig 4A, relatively more of Ssa1-T175N and Ssa1-D158N was eluted as compared to wt Ssa1, suggesting that the mutant Ssa Hsp70s interacted more strongly with Ydj1. The eluted fractions from Ydj1 bound beads were further probed with an anti-Hsp90 antibody. A similar amount of Hsp90 was eluted from strains expressing wt or mutant Ssa Hsp70 isoforms (Fig 4A).

We further monitored Ydj1 interaction with wt Ssa1 and its mutant versions using Bio-layer Interferometry (BLI) as described in Materials and Methods. BLI, an optical label-free method based upon changes in the interference pattern of light, is extensively used to monitor biomolecular interactions in real-time. The purified Ydj1 was immobilized on a biosensor surface, and Ssa Hsp70 was used at varying concentrations as an analyte in a solution containing 5mM ATP. As shown in Fig 4B (S4 Dataset), incubation of a Ydj1-immobilized biosensor tip with a solution containing Ssa1, Ssa1-T175N or Ssa-1D158N led to an increased BLI response. Furthermore, at similar concentrations of Hsp70s, the binding response was much stronger for Ssa1-T175N or Ssa1-D158N than wt-Ssa1, suggesting that Ydj1 affinity is higher for Ssa1-T175N and Ssa1-D158N as compared with wt-Ssa1, which is in agreement with the above pulldown assay showing relatively stronger binding of Ydj1 with Ssa1-T175N or Ssa1-D158N. Further Ssa1-T175N displayed a stronger binding than Ssa1-D158N to Ydj1.

### The Ssa1-T175 and Ssa1-D158 mutations are proximal to the Ydj1 binding site

**Fig 5:**
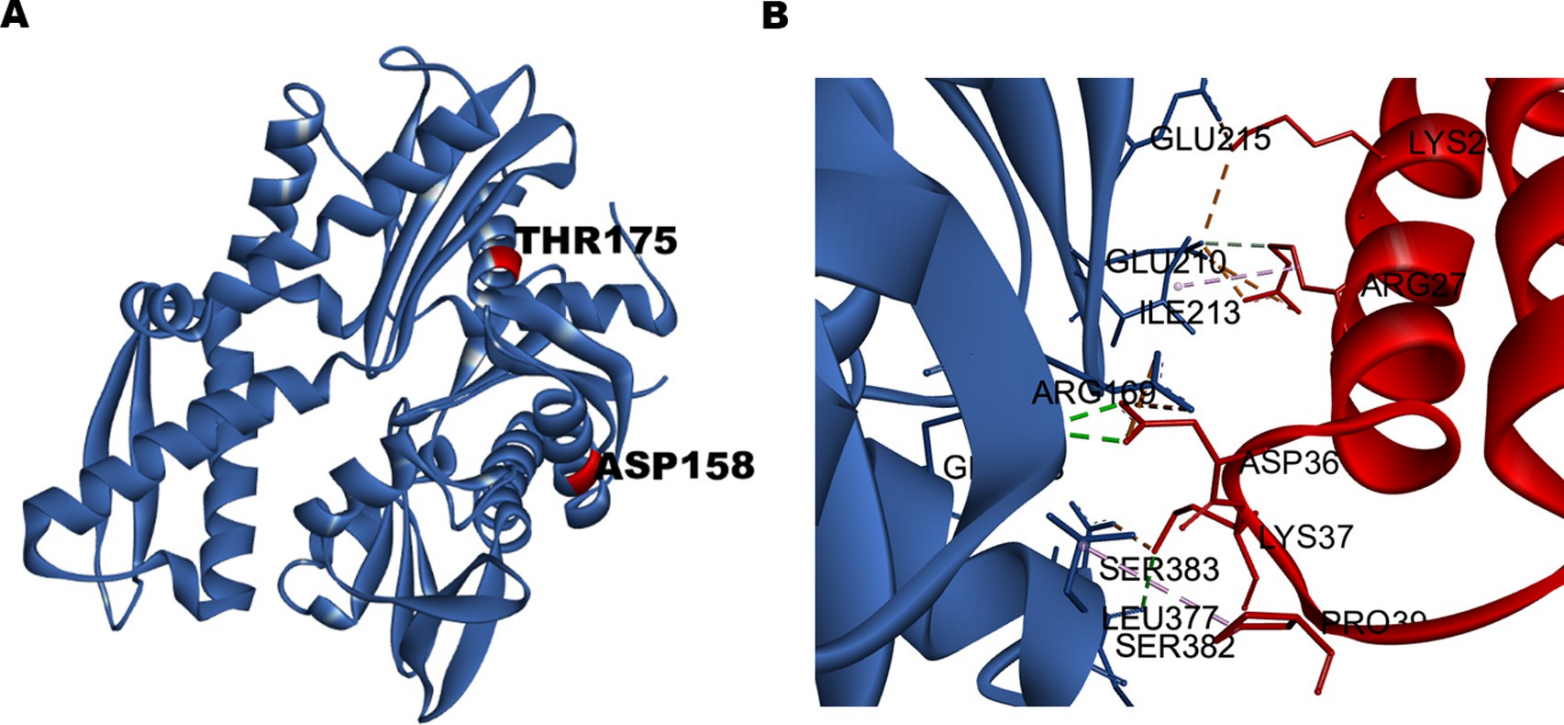
**(A)** The structure of NBD of yeast Hsp70 extracted from the complete structure of Hsp70 obtained from AlphaFold Protein Structure Database. **(B)** The interacting residues of Hsp70 (blue) and Hsp40 (red) docked complex.

The data presented show that the T175 and D158 residues influence Ydj1 interaction to Ssa Hsp70. Both residues lie in the NBD of Ssa Hsp70. As Hsp40 proteins primarily interact with their J-domain to Hsp70s, we modelled the complex of the J-domain of Ydj1 (J-Ydj1) with NBD of Ssa Hsp70 (NBD-Ssa1) to identify whether the T175 and D158 residues lie at, or near to, the binding site of Ydj1. We extracted the NBD region from the 3D structure of Ssa1 Hsp70 from *S*.*cerevisiae* (shown in Fig 5A). S7 Fig shows the alignment between NBD of *S*. *cerevisiae* Ssa1 and *E*. *coli* DnaK. The NBD of Ssa1 was docked to the J-domain of Ydj1 (pdb ID 5VSO) using the HDOCK protein-protein docking webserver [36]. As expected from previous studies, we found interaction between D36 in the HPD motif of J-Ydj1 with R169 of NBD-Ssa1 (Figs 5B and S8B). Additional interactions observed between the J-Ydj1 and NBD-Ssa1 from protein-protein-docked complex are mentioned in Table 2. The docked complex of Ssa1 NBD and the J-domain of Ydj1 were superimposed onto the available *E*.*coli* DnaK-J domain of the DnaJ complex (pdb id 5NRO) with RMSD of 0.475 Å (S8A Fig). As shown in S8B Fig, the modelled structure of the NBD(Ssa1)-J(Yjd1) complex shows that the T175 and D158 residue of DnaK lie near to the Ydj1 binding site.

**Table 2:** The list of interactions observed in the docked complex of yeast Ssa1-Ydj1 J-domain.

Residues in Ssa1	Residues in Ydj1	Distance (angstrom)	Type of interaction
ARG169	ASP36	2.98	Electrostatic
ARG169	ASP36	4.78	Electrostatic
GLN373	ASP36	2.97	Hydrogen Bond
GLN373	ASP36	2.88	Hydrogen Bond
ILE213	ARG27	4.55	Hydrophobic
GLU210	ARG27	2.94	Hydrogen Bond
GLU210	ARG27	2.80	Salt Bridge
GLU210	ARG27	4.37	Electrostatic
GLU210	LYS23	3.83	Salt Bridge
GLU215	LYS23	2.63	Salt Bridge
SER383	LYS37	2.71	Salt Bridge
SER382	LYS37	2.91	Hydrogen Bond
LEU377	PRO39	4.95	Hydrophobic

### Asn substitution at T175 or D158 in Ssa1 affects Hsp90 interaction

**Fig 6:**
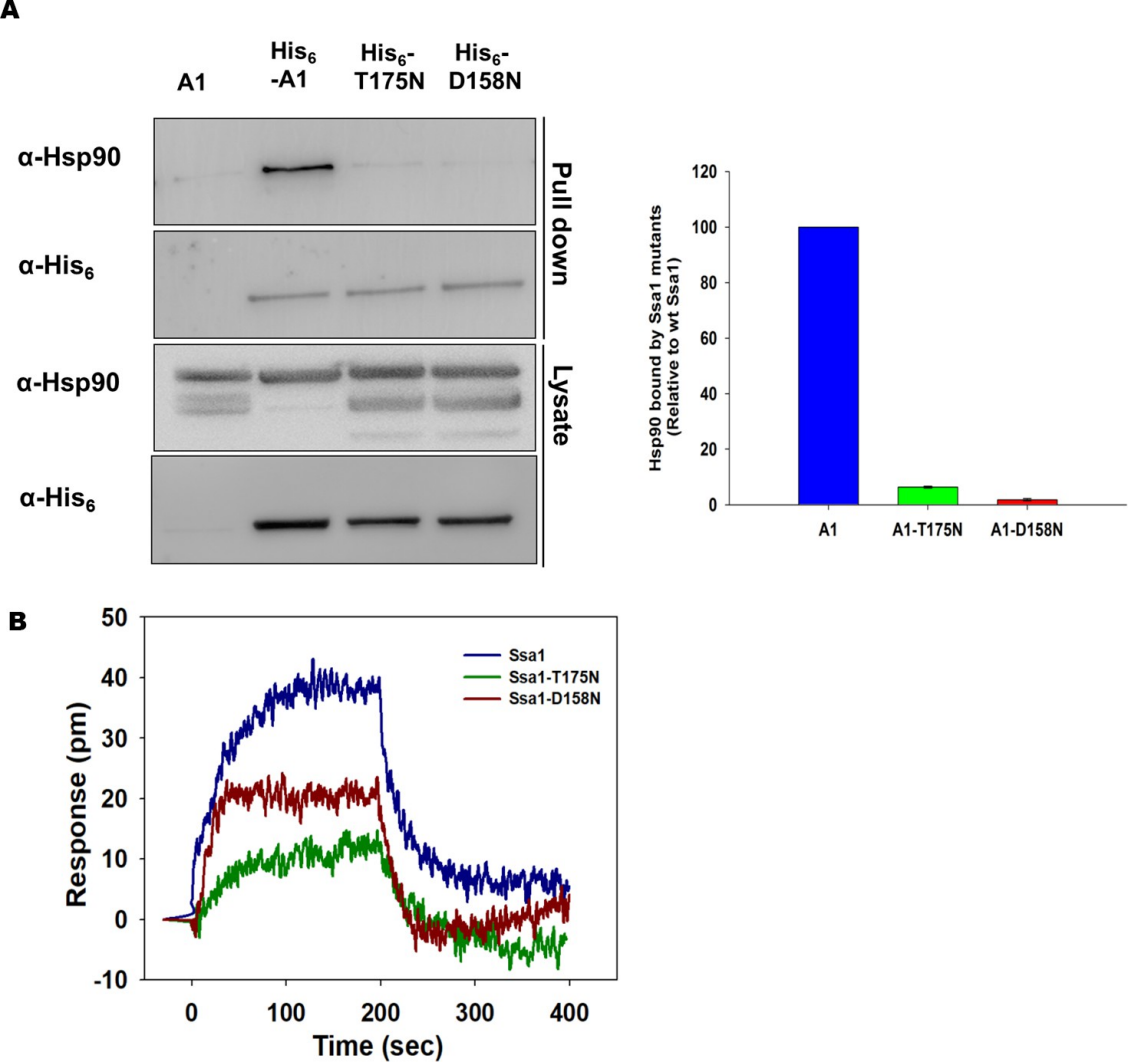
Ssa1-T175N and Ssa1-D158N show weaker interaction with Hsp90. **(A)** Equal amount of yeast lysate was incubated with cobalt metal affinity resin. The bound proteins were eluted with buffer containing 300mM imidazole and probed with anti-His_6_ and anti-Hsp90 antibodies. **(B)** Hsp70 (wt-Ssa1, Ssa1-T175N or Ssa1-D158N) and Hsp82 interaction was monitored using BLI. Kinetic association and dissociation were monitored using 1.0μM of Hsp70 with Hsp90 immobilized on biosensor. Green, red, and blue curves show interaction of Ssa1-T175N, Ssa1-D158N and wt-Ssa1 respectively with Hsp90. Error bars represent standard error from 3 different biological replicates.

We further examined Hsp70-Hsp90 interaction in cells expressing wt or mutant Ssa1. The cells expressing His_6_-tagged wt or mutant Ssa1 Hsp70s were grown until an O.D._600nm_ ~1.0. The cells were collected, and cellular lysates were incubated with Co^2+^-NTA beads to capture His_6_-tagged Ssa Hsp70. The beads were washed, and bound proteins were eluted. The eluted fractions were probed with anti-Hsp90 or anti-His_6_ antibodies on immunoblots. As seen in Fig 6A (S5 Dataset), although similar levels of Hsp70s were detected, Hsp90 levels were found to be significantly reduced in cells expressing mutant Ssa Hsp70s, as compared to those expressing wt Ssa1. These results suggest that the mutant Ssa Hsp70s, compared to wt Ssa1, bind at relatively lower affinities to Hsp90. Similarly, we examined the interaction between purified His_6_ tagged Sti1 with Hsp70 and Hsp90. We found no difference between interaction of Sti1 with both Hsp70 and Hsp90 in A1 and A1-T175N expressing yeast strains, suggesting that loss of Hsp70 and Hsp90 interaction is not due to Sti1 (S9 Fig).

Next, we examined whether the Ssa1 mutant proteins, defective in their interaction with Hsp90 in vivo, also exhibited similar defect in vitro. To examine this, His_6_-Ssa1, His_6_-Ssa1-T175N, His_6_-Ssa1-D158N, and Hsp82 were purified, and interaction between Ssa Hsp70 and Hsp90 was monitored using BLI. Hsp82 was immobilized onto a CM5 biosensor and immersed into a solution containing wt-Ssa1, Ssa1-T175N or Ssa1-D158N as analyte. The binding was monitored as an increase in the BLI response (Fig 6B) (S6 Dataset). As seen, at the equilibrium phase of association, the BLI response for Hsp82 to wt-Ssa1 interaction was about 2 and 4 fold higher than that of Ssa1-D158N and Ssa1-T175N, respectively. Overall these results suggest that T175N and D158N position in Ssa1 is crucial for Hsp90 interaction.

### Asn substitution at T175 or D158 in Ssa1 triggers temperature sensitivity

Since the mutant Ssa Hsp70s interacted poorly with Hsp90s, and as Hsp70 is required for Hsp90 function, we wondered if disruption of Hsp70-Hsp90 interaction might have an effect on cell growth under suboptimal conditions requiring increased activity of the chaperones. To examine the effect of Ssa1-D158N and Ssa1-T175N on their ability to support essential functions required for cellular viability, we compared the growth of the A1-D158N and A1-T175N strains with that of A1 at different temperatures. Cells were grown in liquid YPAD media at 30°C, and further serially spotted onto solid growth media. The cellular growth was monitored at 16°C, 30°C and 37°C. As evident from S10A Fig, the A1-D158N strain grew similarly to the wt A1 strain at 30°C however, partial growth defect was observed at both 16°C and 37°C. As compared to A1, the A1-T175 strain showed poor growth at 16°C, and a severe growth defect at 37°C. Further, we compared the growth rate of the A1, A1-T175N and A1-D158N strains at 30°C and 37°C in liquid YPAD media. Similar to as seen above, we found that the A1-T175N strain grew slowly at 30°C compared to the A1 and A1-D158N strains (S10B Fig and S18 Dataset). We did not observe any growth of the A1-T175N strain at 37°C (S10C Fig and S19 Dataset). Overall, these results suggest that cells expressing the Ssa1 mutants grow poorly at suboptimal temperatures and that Ssa1-T175N is unable to support growth at higher temperatures (37°C).

The relatively poor growth of A1-T175N and A1-D158N at suboptimal temperatures could be due to lack of activation of protective stress responses in these strains. To monitor stress response, we transformed cells with plasmid encoding HSE-*lacZ* that expresses β-galactosidase from promoter containing heat shock elements (HSE). Under stress, the heat shock factors (Hsf1) bind to HSE resulting in transcriptional activation of downstream genes. The transformants were first grown in selective liquid SD media at 30°C, and further incubated at 37°C for 4 hours to induce heat shock response. An equal number of cells were then used for β-galactosidase assays as described in Materials and Methods. As shown in S10D Fig (S20 Dataset), as compared to A1, both A1-D158N and A1-T175N showed about a 2 fold higher β-galactosidase activity indicative of an elevated heat shock response in these strains.

The growth defect observed for A1-D158N and A1-T175N at suboptimal temperatures, as well as a higher heat shock response, could be be due to an inability of the cellular proteostasis machinery to support folding of protein substrates involved in essential processes. Therefore, we monitored reactivation of in-vivo expressed thermolabile firefly luciferase after its denaturation at higher temperature. Luciferase is a widely used substrate of Hsp70. The cells expressing firefly luciferase were grown at 30°C until mid-exponential phase and subsequently shifted to 48°C for 30 minutes. The refolding of the thermally-denatured luciferase was initiated by recovering cells at 30°C and monitoring, luciferase activity by measuring increase of luminescence after 60 minutes. The luciferase activity post heat shock was compared with that obtained before heat treatment. Since, Hsp104 is essentially for disaggregation, we used an *hsp104*Δ strain to monitor basal refolding of denatured luciferase. As expected, *hsp104*Δ cells showed only poor luciferase refolding (S10E Fig) (S21 Dataset). The A1 strain showed about 12 fold better luciferase refolding as compared to *hsp104*Δ. The luciferase reactivation in A1-T175N and A1-D158N was found to be about 2 and 6 fold respectively, lower as compared to that in A1 suggesting that as compared to wt Ssa1, both mutants were partially defective in luciferase refolding *in vivo*.

### Sti1 is indispensable for optimal growth in cells carrying the A1-T175N and A1-D158N mutations

Hsp70 interacts with Hsp90 directly through its nucleotide-binding domain and indirectly through the bridge protein Sti1 [11,16,17]. Deletion of Sti1 is known to impair the maturation of Hsp90 clients such as v-Src (and thus reduce v-Src toxicity), likely by affecting Hsp90 functions or the transfer of clients from Hsp70 to Hsp90. Since our data suggest that D158N and T175N residues influence Ssa1 interaction with Hsp90, we wondered whether the observed reduction of v-Src toxicity in the A1-T175N or A1-D158N strain was due to disruption of Hsp70-Hsp90 interaction via Sti1 or the nucleotide-binding domain. To approach these possibilities, we attempted to examine the effect of a Sti1 deletion in the A1-T175N and A1-D158N strains on v-Src toxicity. If the reduced toxicity in strains expressing the mutant Hsp70s is due to disruption of Sti1 mediated Hsp70-Hsp90 interaction, the lack of Sti1 in these strains should not have an additive effect on the cellular growth upon v-Src overexpression.

**Fig 7:**
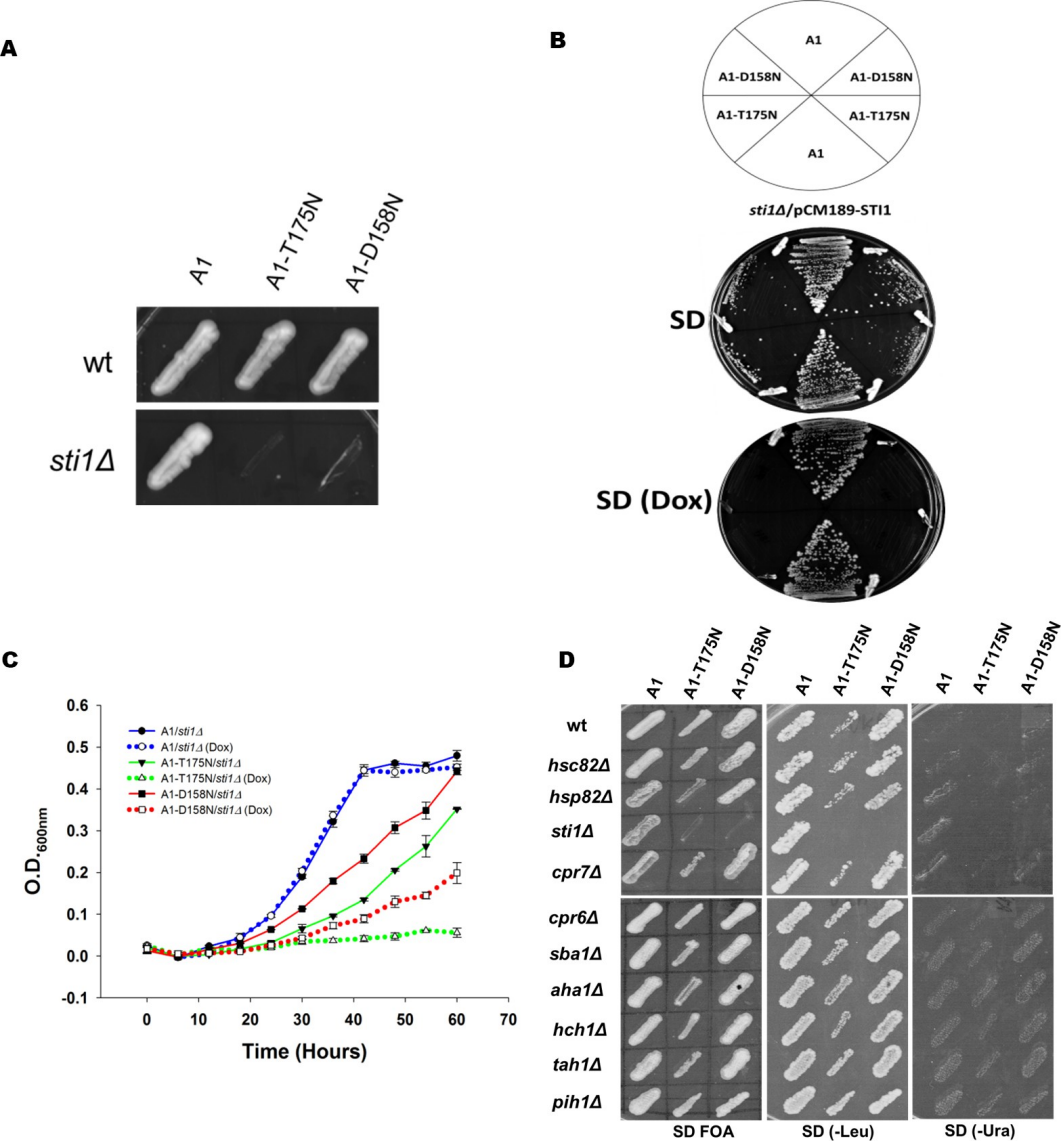
*Sti1* is indispensable for optimal growth of A1-T175 and A1-D158. **(A)** A1-T175N and A1-D158N cells haboring plasmid encoding Ssa2 were patched onto selective SD media with and without FOA. Shown is growth after 5 days of incubation at 30°C. **(B)***sti1Δ* deletion strains with wt A1 and its mutants were transformed with pCM189-STI1. Growth was monitored onto SD and SD (Dox) plates for 5 days. **(C)** 5–6 transformants were pooled and grown in selective liquid SD media. Cells were further sub cultured at 0.02 O.D._600nm_ in SD or SD (Dox) media at 30°C. **(D)** Effect of deletion of various chaperones and co-chaperones on the growth of wt, A1-T175N and A1-D158N. Error bars represent standard error from 3 different biological replicates.

We thus attempted to delete *STI1* in A1, A1-T175N and A1-D158N as described in Materials and Methods. Interestingly, while *STI1* could be deleted in the A1 strain, a similar knockout was not possible in A1-T175N and A1-D158N. We further attempted to shuffle a plasmid encoding Ssa1 in a strain lacking Sti1 (SY289) with one encoding either of the Ssa mutations (pRS315-SSA1, pRS315-SSA1-T175N or pRS315-SSA1-D158N). The strain SY289 expressing wt Ssa2 from a *URA3*-containing plasmid was transformed with a *LEU2*-based plasmid encoding wt or mutant Ssa1 Hsp70s. The transformants were patched onto solid SD media containing fluoroorotic acid (FOA). The cells were further replicated onto SD media lacking leucine. As seen in Fig 7A, whereas cells expressing wt Ssa1 showed normal growth onto solid SD media containing FOA and lacking leucine, no growth was visible onto FOA for *sti1*Δ cells harboring plasmids encoding either Ssa1-T175N or Ssa1-D158N. These results suggest that the Ssa1 mutant alleles, when present as the sole source of Ssa Hsp70, are unable to support cell growth in the absence of Sti1.

To further confirm whether Sti1 is required for cellular growth of A1-T175N and A1-D158N or not, we constructed A1, A1-T175N and A1-D158N strains, expressing Sti1 from a plasmid-borne gene under the control of a doxycycline-repressible promoter, and lacking chromosomally encoded Sti1. The cells were grown onto solid SD growth media with and without doxycycline. As seen in Fig 7B, though all strains grew in the absence of doxycycline, only those with mutant Ssa1 failed to grow in the presence of the repressor. Similar results were observed when cells were grown in the presence and absence of doxycycline-containing liquid SD media (Fig 7C) (S7 Dataset). We found that in the absence of Sti1 expression, the A1-D158N strain grows only partially in liquid growth media, and no increase in growth was observed for the A1-T175N strain. Collectively, these results show that Sti1 is required for optimal growth in cells expressing A1-T175N or A1-D158N as a sole source of Ssa Hsp70.

In addition to Sti1, Hsp90 requires the assistance of various other co-chaperones. While co-chaperones such as Sti1 and Aha1 modulates Hsp90 ATPase activity, others such as Cpr7, Cpr6, Hch1, Tah1, Sba1, and Pih1 modulate conformational transition of Hsp90 during various stages of its reaction cycle [9,10,15]. We thus examined whether the observed effect on cell growth in A1-T175N and A1-D158N is specific for Sti1 or more general to any of the other Hsp90 co-chaperones. The gene encoding the desired Hsp90 co-chaperone in A1 was replaced with the gene encoding KanMX4. The knockout strains were transformed with a plasmid encoding for Ssa1-T175N or Ssa1-D158N. 3–4 transformants were then pooled and patched onto solid SD media containing FOA to shuffle out a wt Ssa1 encoding plasmid. The FOA plate was then replica-plated onto solid SD media lacking leucine or uracil. As seen in Fig 7D, wt Ssa1 supported normal growth in the absence of either of the Hsp90 isoform or any of their co-chaperones. The cellular growth in *cpr7*Δ, *sti1*Δ and *hsp82*Δ was relatively slow as compared to other knockout strains. As expected, *sti1*Δ did not support growth in any of the strains expressing the mutant Ssa Hsp70s. For A1-T175N, cells possessing both Hsp90 isoforms, one of the Hsp90 isoforms, or deletions of Hsp90 co-chaperones, except *sti1*Δ grew poorly. The absence of either of Hsp90 isoforms or any other Hsp90 co-chaperone other than Sti1 in A1-D158N had no significant effect on cellular growth. Overall, these results show that the growth defect observed in A1-T175N and A1-D158N is specific for *sti1*Δ and not general for other Hsp90 co-chaperones.

Structurally, Sti1 comprises of 3 TPR domains (TPR1, TPR2A and TPR2B), and two aspartate- and proline-rich domains (DP1 and DP2). The TPR domains, TPR1 and TPR2 bridge Hsp70 and Hsp90 by interacting at EEVD and MEEVD residues present at their C-terminus, respectively [12]. Though the exact role of the DP1 and DP2 domains is not clear, they have been shown to promote client activation in vivo. To further elucidate Sti1 functions in the A1-T175N and A1-D158N strains, we examined the ability of various Sti1 derivatives to complement Sti1’s role in supporting cell growth in these mutants. S11A Fig shows schematics of various Sti1 derivatives that were examined to support cell growth in the absence of Sti1 in cells expressing Ssa1-T175N or Ssa1-D158N as sole Ssa Hsp70. The derivatives either lack the TPR region required for interaction with Hsp70 or Hsp90, or the linker region that facilitates conformational changes in Sti1. The genes encoding these derivatives were subcloned under control of the GPD promoter and transformed into A1, A1-T175N or A1-D158N lacking Sti1 and harboring a *Ura3*-containing plasmid encoding Ssa2 (S11A Fig). A pool of 5–6 transformants was patched onto solid media with and without FOA to examine the growth of cells without wt Ssa2. As shown in S11B Fig, A1 cells expressing wt Sti1 or any of its derivatives showed optimal growth onto solid media containing FOA. When complemented with wt Sti1, A1-T175N and A1-D158N grew similar onto media with and without FOA. When complemented with a Sti1-derivative lacking linker region, the A1-T175N and A1-D158N displayed relatively slow growth on growth media with FOA. Other Sti1 derivatives expressing only DP2 or TPR2B or TPR2A-TPR2B-DP2 could not complement Sti1 function in supporting growth in cells expressing mutant Ssa1 as sole Ssa Hsp70. Similarly, Sti1 derivatives lacking region DP2 or TPR2B-DP2 did not support growth in *sti1Δ* A1-T175N and A1-D158N strains. These results suggest that full-length Sti1 is important to support optimal cells growth, and that the function of the linker region is not crucial for the growth phenotype observed in cells expressing either of mutant Ssa1s as a sole source of Ssa Hsp70 (S11B Fig).

### Sti1 is required for the maintenance of proteastasis in the A1-T175N strain

**Fig 8:**
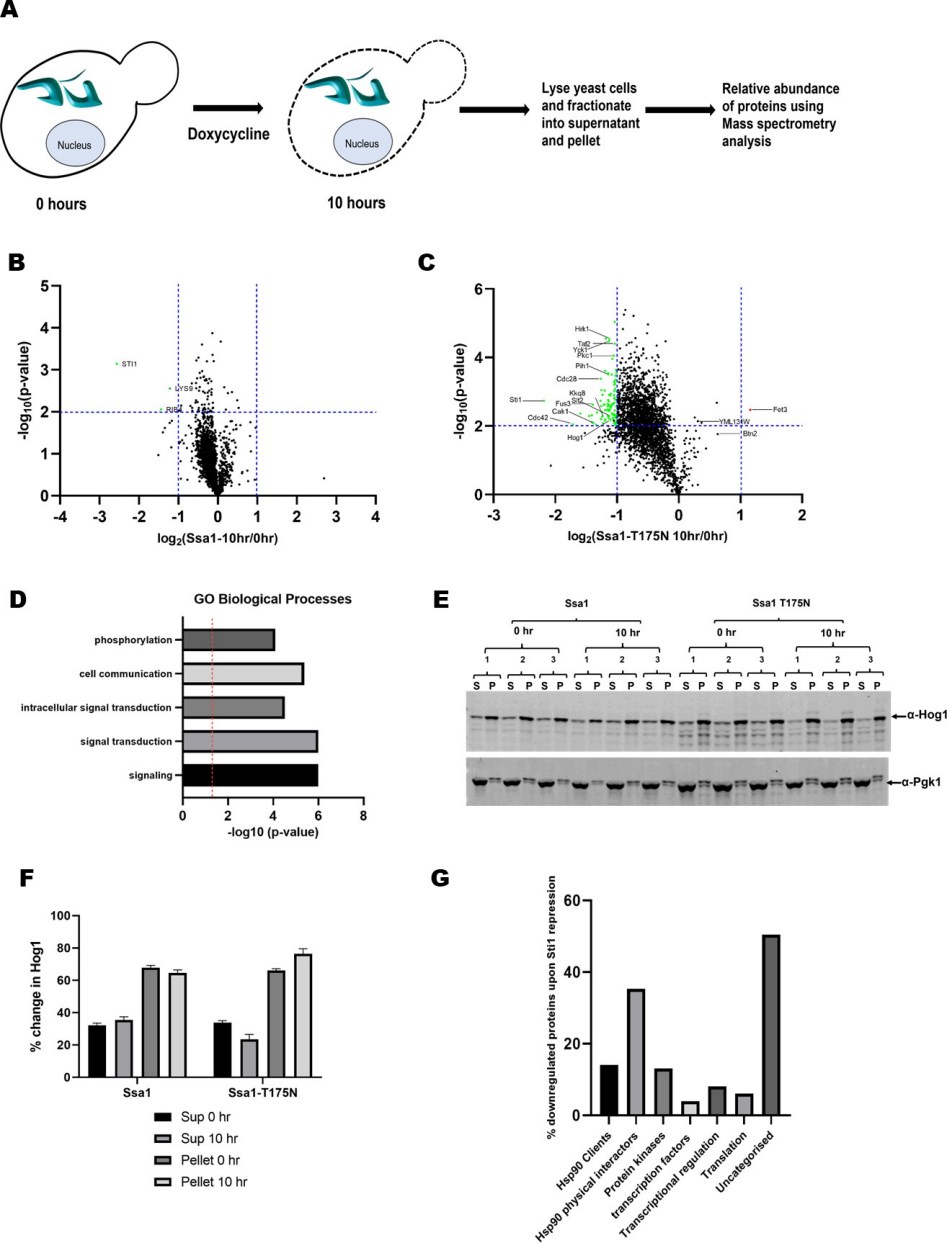
Sti1 is required for maintenance of Hsp90 clients in A1-T175N strain. **(A)** Schematics representing the experimental design. Cells were harvested post 10 hours of Sti1 repression. Cells (at 0h and 10h) were lysed and fractionated into supernatant and pellet fractions. Supernatant was used for mass spectrometry based proteomic analysis. **(B)** Volcano plot showing the log_2_ fold of proteins upon Sti1 repression in A1 strain **(C)** Volcano plot showing the log_2_ fold of proteins upon Sti1 repression in A1-T175N strain. 98 proteins were downregulated upon Sti1 repression (green dot). Only one protein was found to be upregulated by more than 2 fold (red dot). Significant cut-off was set for >2 fold and p<0.01 (N = 3, p<0.01). **(D)** GO term analysis shows the enriched proteins involved in various biological processes. Red dotted line shows p<0.05. **(E)** Western blot showing the Hog1 abundance upon Sti1 repression (N = 3). **(F)** Quantification of Hog1 in supernatant and pellet fractions. Percentage fractions of Hog1 in supernatant and pellet were estimated with respect to total (100% = supernatant+pellet) Hog1 protein in A1 and A1-T175N strain. **(G)** Significantly downregulated proteins in different biological processes and Hsp90 physical interactors. Error bars represent standard error from 3 different biological replicates.

The strain expressing Hsp70 mutant Ssa1-T175N shows increased aggregation of v-Src and an elevated heat shock response, suggesting perturbations in proteostasis. We thus carried out a proteome-wide analysis to examine the fate of cellular proteins in the A1-T175N strain. Fig 8A describes the schematics of the TMT-based mass spectrometry study utilized to explore the proteome. The changes in the abundance of proteins in the soluble fraction of cellular lysates were examined upon repression of Sti1 expression post 10h (S12A and S12B Fig) (S22 Dataset) and compared with that before repression (0h). The mass spectrometry study showed not many changes in solubility of most of the proteins in A1 strain (Fig 8B and S2 Excel sheet). The mass spectrometry identified 3166 proteins in the supernatant fraction of which 2691 were used for further analysis as the rest of the proteins showed significantly lower abundance. Proteins which showed >2 fold difference in abundance (10h versus 0h fraction) were considered to be significantly altered. Based on this criteria, we observed lower abundance of a major fraction (3.7%) of proteins in the soluble fractions upon Sti1 repression. Among them, a majority was found to be kinases and transcription factors indicating altered Hsp90 activity (Fig 8C and S1 Excel sheet). GO term analysis showed that pathways involved in signalling, signal transduction, and protein phosphorylation are markedly downregulated in the Ssa1-T175N background upon repression of Sti1, which is as expected from a compromised Hsp90 activity (Fig 8D). Many of the kinases, such as Ypk1, Slt2, Yck2, and Cdc28 involved in Ser/Thr phosphorylation and cell cycle control were found to be decreased upon Sti1 repression in the A1-T175N mutant strain [37–42]. Repression of Sti1 in A1-T175N also reduced the abundance of various transcription factors, such as Taf2, Taf9, Brf1, and DNA Polymerase subunits such as Pol12 & Pol31 as well as various ribosomal proteins such Rpl24A, Rpl24B, Rpl23A, Rpl38 and Rps1B [43–46]. We also found a reduced abundance of Cog6 (49.1%), Vps45 (49%), Vps1 (48.7%), Sft1 (41.5%) and Erv25 (48.9%) in the soluble fraction which are critical component for vesicular trafficking. The decreased abundance of Conserved Oligomeric Golgi (Cog6) may result in defect in ER to golgi trafficking, vesicular tethering and IPOD formation [47,48]. Vps45 is essential for vacuolar protein sorting and its lower abundance results in a defect in vesicular trafficking to endocytic pathways and reduced iron uptake in vacuoles [49]. Similarly, depletion of Erv25 results in an increase of the unfolded protein response (UPR) [50]. We found serine/threonine kinases (S/T kinase), such as Pkc1 involved in the maintenance of cell wall integrity, to be significantly depleted in the soluble fraction. Similarly, the inactivation of kinases Hrk1 and Mck1 is known to result in loss of ion homeostasis by lower activation of Pma1 on cellular membrane [51,52]. Cdc28, Cak1 and Cdc48 that are known to regulate cell cycle were also found to be down-regulated upon Sti1 repression in A1-T175N strain. Their inactivation is known to result in failure of both meiosis and mitosis and leads to cell cycle arrest. Cdc48 is also involved in retrograde transport of proteins, ERAD and membrane fusion [53,54]. Depletion or mutation in Cdc48 leads to ER stress and also increased cell death due to apoptosis [55]. We further analysed the modulation of Hsp90 interactors and found that about 35% of those interactors were downregulated. Similar analysis with proteins involved in different biological functions showed that various kinases (~13%) and Hsp90 clients (~14%), were also downregulated suggesting that Hsp90 functions are compromised in A1-T175N (Fig 8G and Table 3 and S9 Dataset). Overall the proteome-wide analysis showed that depletion of Sti1 in cells expressing Ssa1-T175N leads to large scale alterations in the proteome which could be due to altered effect on Hsp90 activity upon mutations in Ssa1-T175N. We had also checked the solubility in A1 upon Sti1 repression. We plotted volcano plot. We could only observe 2 proteins which were significantly downregulated upon Sti1 repression (Fig 8B and S2 Excel sheet). A previous study [18] have shown the enrichment of stress response and protein folding/refolding processes upon Hop/STI1P knockout in cell lines. We could not observe such changes in our experimental set up due to differences in suppression of Sti1. Nevertheless, this indicates the specificity of decrease in solubilty is related to Ssa1-T175N mutant only.

**Table 3:** List of downregulated Hsp90 interactors.

Protein	Functions
TAF2	Transcription Factor
TAF9	
ADR1	
BRF1	
Med1	Transriptional regulation
Hog1	
Fpr3	
Adr1	
Dcs1	
Sap30	
PIH1	
CDC28	
SUP35	Translation
RPS1B	
RPL24B	
RPL23A	
RPL38	
RPL24A	
NRK1	Protein Kinases
Pkc1	
Ypk1	
Hog1	
HRK1	
SLT2	
YCK2	
KKQ8	
MCK1	
CDC28	
SFT1	
CAK1	
FUS3	
COG6	Hsp90 clients
TDH1	
ROD1	
PKC1	
ADR1	
YPK1	
HOG1	
SLT2	
YCK2	
VPH1	
URA2	
MCK1	
CDC28	
FUS3	
FET3	Hsp90 physical interactors
GUT2	
FIS1	
YPR127W	
TDH1	
ERV25	
PSE1	
SUP35	
VPS1	
MDH2	
PET10	
AYR1	
FPR3	
CTT1	
YOP1	
RTN2	
ADR1	
DCS1	
HOG1	
SLT2	
IML2	
PST2	
PST1	
YET3	
VPH1	
PIH1	
URA2	
FMP16	
RPL38	
DPP1	
SDS24	
RPL24A	
MCK1	
PUT1	
POR1	
STI1	

To further examine the altered solubility of Hsp90 clients, we examined the abundance of one of the Hsp90 substrates, Hog1 (Mitogen-activated protein kinase) in both cellular lysate supernatant and pellet [56]. The protein level was monitored before and after Sti1 repression post 10h. As seen, the decrease of Hog1 levels in the soluble fraction and increase in the pellet fraction after Sti1 repression was relatively more in A1-T175N compared to A1 (Fig 8E and 8F) (S8 Dataset). Overall, these results suggest an important aspect of Hsp70-Hsp90 direct interactions, mediated by the nucleotide-binding domain, in promoting solubility of the proteome including diverse pathways such as transcription, translation, organellar specific protein sorting, cell cycle control, stress responses and protein degradation.

### Ydj1 assists Ssa1-T175N and Ssa1-D158N better than wt Ssa1 in luciferase refolding activity

The Hsp40 Ydj1 assists Hsp70 function by stimulating ATPase activity as well as substrate transfer [22,57]. The above pull-down and BLI studies show that Ydj1 interaction with Ssa1 mutant proteins is relatively stronger than with wt Ssa1. We thus examined whether this increased affinity of Ydj1 with Ssa1 mutants also affected their protein refolding activity. The unfolded heat-denatured luciferase is an obligate Hsp70 substrate for its refolding. The refolding of luciferase is further enhanced when Hsp90 is co-incubated with Hsp70s in a refolding buffer [58].

**Fig 9:**
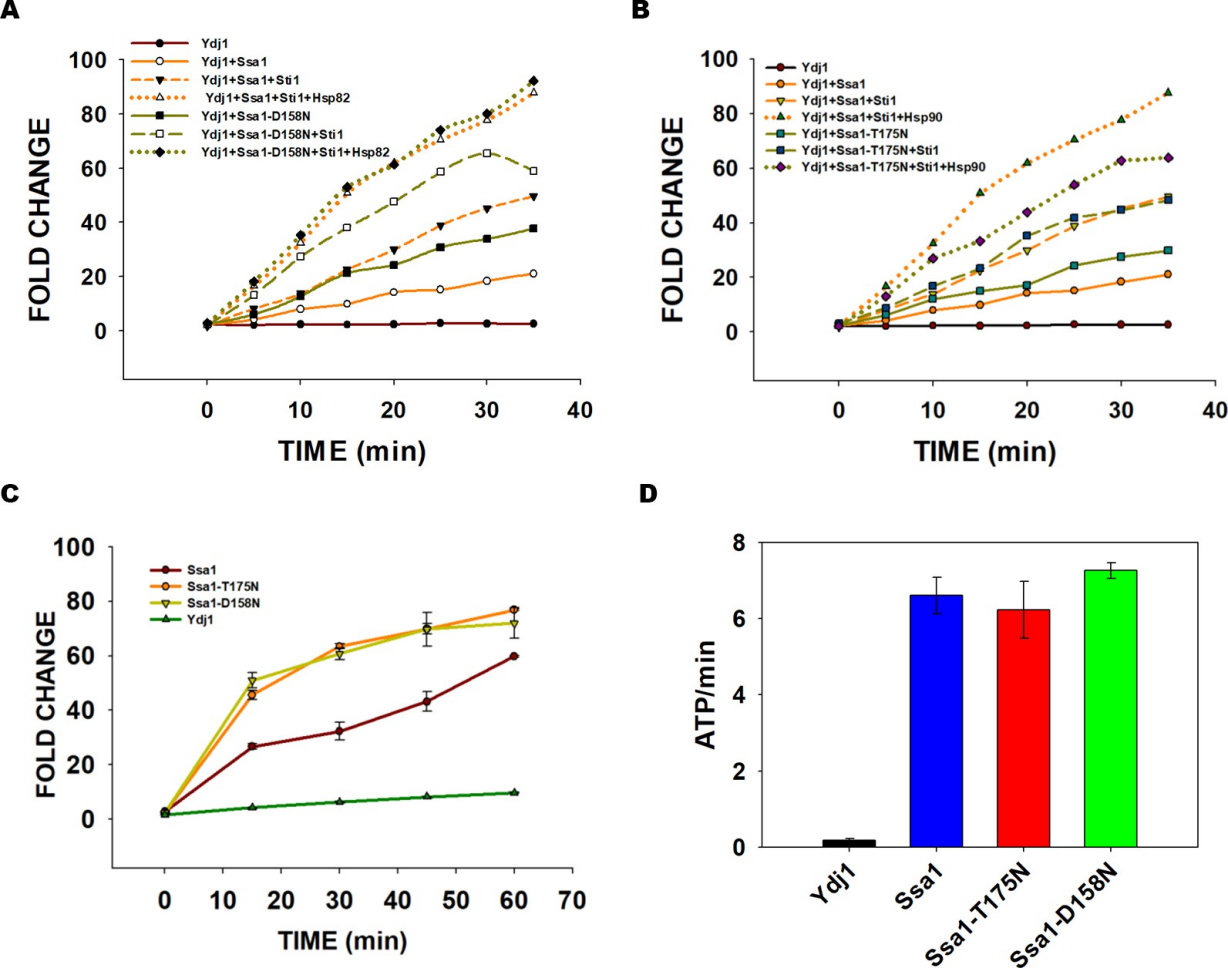
**The fold increase in luciferase activity by Hsp90 is lower with Ssa1-T175N and Ssa1-D158N as compared with wt Ssa1: (A)** Luciferase (80nM) was denatured in the presence of 1mM ATP at 45°C for 10 min. The denatured luciferase (40nM) was incubated in presence of 0.5μM Hsp70 (Ssa1, Ssa1-T175N or Ssa1-D158N) and 0.3μM Ydj1. The reaction was incubated at 25°C, and refolding was initiated by the addition of 1mM ATP. **(B)** Ydj1 mediated stimulation of ATPase activity of Ssa1-T175N and Ssa1-T158N with respect to that of wt-Ssa1. **(C, D)** The denatured luciferase (40nM) was incubated in the presence of 0.3μM Ydj1, 0.5μM Hsp70 (Ssa1, Ssa1-T175N or Ssa1-D158N), 2.5μM Sti1 and 0.9μM Hsp82, and the refolding was monitored as described above. Error bars represent standard error from 3 different biological replicates.

The luciferase was denatured upon incubation at 45°C for 7 min, and the refolding was initiated by incubating denatured luciferase with Ssa1, Ssa1-T175N or Ssa1-D158N in the presence of Ydj1 at 25°C for different time intervals. As seen in Fig 9C(S12 Dataset), Ydj1 alone is unable to refold heat-denatured luciferase. As seen by an increase in luminescence, the fraction of refolded luciferase increased when Ssa1, Ssa1-T175N or Ssa1-D158N was added into a refolding buffer containing Ydj1. The refolding increased in a time-dependent manner and nearly saturated at about 30 min. At all incubation times, the increase in luminescence was relatively more pronounced with Ssa1-T175N or Ssa1-D158N than with wt-Ssa1. After 30 min of incubation, luciferase refolding was about 60-fold in the reaction containing Ssa1-T175N:Ydj1 or Ssa1-D158N:Ydj1 while it was only 30-fold with Ssa1:Ydj1. Overall, the data suggest that in the presence of Ydj1, Ssa1-T175N or Ssa1-D158N are more efficient than Ssa1 in refolding luciferase in vitro. We further examined the effect of Ydj1 on Ssa1 and its mutant’s ATPase activity. As seen in Fig 9D (S13 Dataset), in the presence of Ydj1, ATPase activities of Ssa1, Ssa1-T175N and Ssa1-D158N were similar.

We further examined how Hsp90 cooperated with Ssa1 mutant proteins in the refolding of denatured luciferase. The Hsp82 isoform of Hsp90 was added to the refolding reaction containing either wt Ssa1 or its mutants, Ydj1, and Sti1 as bridge protein between Hsp70 and Hsp90.

As expected, the presence of Hsp90 further enhanced luciferase refolding (Fig 9A and 9B) (S10 and S11 Datasets). After 30 min of incubation in the refolding buffer, luciferase refolding was about 80-fold in the reaction containing Ydj1:Sti1:Hsp82 with wt Ssa1 as compared to 60 and 80-fold with Ssa1-T175N and Ssa1-D158N, respectively. The fold increase in luciferase refolding upon addition of Hsp90 and Sti1 in a reaction containing Ydj1:Ssa1(wt or mutant) is 4, 2 and 2 for wt Ssa1, Ssa1-T175N and Ssa1-D158N respectively as compared to reaction containing Ydj1 and different variants of Hsp70 only, suggesting that Hsp90 mediated increase in the substrate refolding is lower with Ssa1 mutants than with wt Ssa1 (Figs 9A and 9B and S13) (S10, S11, S23 and S24 Datasets).

### T175N or D158N mutation in Ssa4, interacts poorly with Ydj1, supports cells growth even in the absence of Sti1

Above data shows that the identified Ssa1 mutant proteins interact relatively strongly with Ydj1, interact poorly with Hsp90, and does not support growth in the absence of Sti1. Our previous study shows that one of the Ssa Hsp70 isoforms, Ssa4 interacts poorly with Ydj1 [26]. In order to examine whether Ssa1-Ydj1 interaction regulates viability of *sti1*Δ cells, we constructed the homologous mutations in the remaining three Ssa Hsp70 isoforms, including Ssa4 and monitored their ability to support cellular survival in the absence of Sti1.

**Fig 10:**
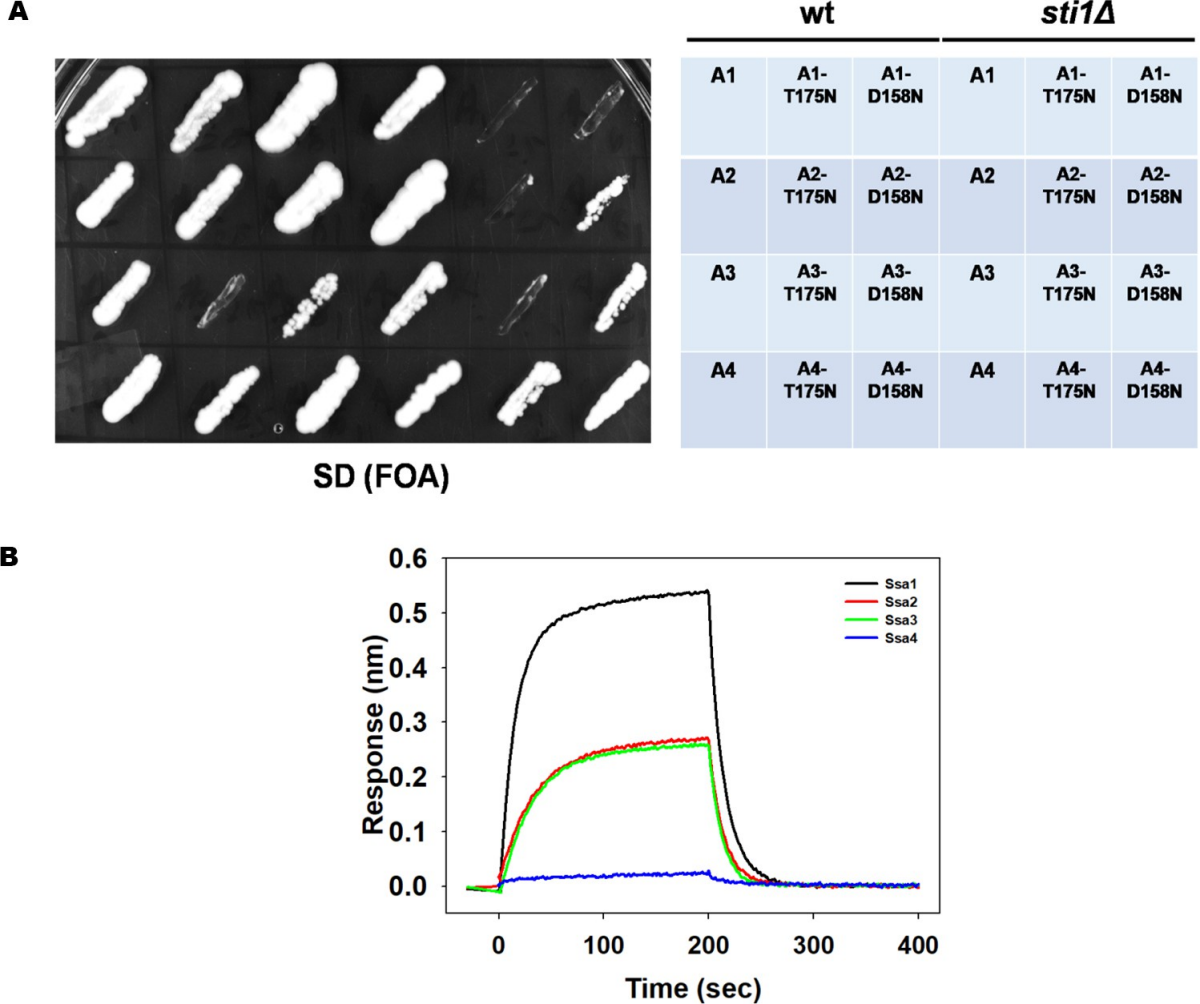
Ssa4-T175N and Ssa4-D158N support cellular growth in absence of Sti1. **(A)** Strains expressing A1, A2, A3 or A4 (or their respective T175N and D158N mutants) in wt and *sti1Δ* background were patched onto FOA plates. Shown here is growth after 8 days of incubation at 30°C. **(B)** Hsp70 (Ssa1, Ssa2, Ssa3 and Ssa4) and Ydj1 interaction was monitored using BLI. Black, Red, Green and Blue represent Ssa1, Ssa2, Ssa3 and Ssa4 respectively.

Ssa2/3/4 were mutated at the position homologous to T175 and D158 in Ssa1 with Asn. The wt and designed alleles (Ssa2/3/4-T175N and Ssa2/3/4-D158N) were expressed from the same Ssa2 promoter as used for Ssa1. The wt strain or that lacking Sti1 harboring a *URA3*-containing plasmid encoding wt Ssa2 as the sole source of Ssa Hsp70 was transformed with *LEU2*-containing plasmid encoding wt or the designed mutant Ssa Hsp70 isoforms. 3–4 transformants were pooled and patched onto FOA containing growth media for counter-selecting the Ssa2-encoding plasmid. As seen, cells expressing wt Ssa Hsp70 isoforms showed optimal growth onto FOA in the presence and absence of Sti1. Though *STI1* cells expressing T175N or D158N mutant allele of Ssa1, Ssa2 or Ssa4 grew well, those carrying Ssa3-T175N/Ssa3-D158N showed significant growth defects (Fig 10A). As expected, cells expressing Ssa1 mutants did not show any growth in the absence of Sti1. Similarly, *sti1Δ* cells expressing Ssa2-T175N and Ssa3-T175N did not grow, while relatively poor growth was observed with Ssa2-D158N and Ssa3-D158N. Interestingly, similar to cells expressing wt Ssa4, those expressing Ssa4 alleles, Ssa4-T175N and Ssa4-D158N, grew well even in the absence of Sti1 (Fig 10A).

We further monitored the interaction of A1, A2, A3 and A4 isoforms of Hsp70 with Ydj1 using biolayer interferometry. As evident in Fig 10B (S14 Dataset), different Ssa Hsp70 isoforms showed varied level of affinities with Ydj1; Ssa1 has the highest, while Ssa2 and Ssa3, both showed similar affinity, followed by Ssa4 which had the least binding affinity to Ydj1.

Overall, the results show that Ssa Hsp70 isoforms, though highly homologous, function differently in the Hsp90 chaperoning pathway, and that the functional distinction is, in part, regulated by their interaction with Ydj1.

## Discussion

Hsp70 and Hsp90 together form a multi-chaperone machinery. The formation and functioning of this dynamic complex are regulated by various co-chaperones. The two chaperones are bridged by Sti1 that also modulates their ATPase activity, and promotes transition among various conformations formed during the reaction cycle [12]. Similarly, Ydj1 modulates the opening and closing of the Hsp70 substrate-binding pocket for client proteins to adopt a form for subsequent processing by Hsp90. The Ydj1 and Hsp90 have been recently shown to compete for the same region on Hsp70 however, the significance of such dynamics is not clear [59]. In the present study, using yeast that express Ssa1 in the absence of other Ssa Hsp70s, we show that a delicate balance exists between the interaction of Hsp70 with Ydj1 versus Hsp90 and any perturbations from such dynamic equilibrium adversely affects Hsp90 function and thus its role in the maintenance of proteastasis.

Hsp70 consists of three functional domains; N-terminal ATPase binding domain, substrate-binding domain and a C-terminal lid. Our mutagenesis study on Ssa1 discovered mutations in the Hsp70 region that inhibited maturation of Hsp90 client proteins v-Src and Ste11. The loss of client maturation suggests Hsp70/Hsp90 network is functionally impaired by the amino acid substitution in the Hsp70 isoform. The two potent Ssa1 alleles unable to support the client maturation had amino acid substitution in the same nucleotide-binding domain. The identified amino acids critical for Hsp90 function were also found to be conserved across species such as in *E*.*coli* DnaK and constitutive Hsp70 isoform (HspA8) in humans (Table 1). Interestingly, among the isolated mutants affecting Hsp90 function, no substitution was observed in the substrate-binding domain of Ssa1. At the C-terminal domain, no single amino acid substitution was observed however, in agreement with role of Sti1, a Ssa1 allele lacking the entire 39 amino acid stretch including Sti1 interaction motif (EEVD) was isolated. Overall these findings suggest that in addition to the well-studied role of the C-terminus of Hsp70 (through Sti1), the Hsp90 function is sensitive to changes in N-terminal domain of Hsp70s.

The reduced client maturation in cells expressing the Ssa1 mutants could be either due to a defect of Hps70 activity or a downstream effect on Hsp90 action. Several lines of evidence suggest that it’s the loss of Hsp90 action that affects client folding in the presence of these Ssa1 mutants as source of Ssa Hsp70. First, all identified mutations show poor affinity with Hsp90. Second, the luciferase refolding with the Ssa1 mutant is higher as compared to wt Ssa1 suggesting that Ssa1 refolding activity is not altered due to mutations. Third, the fold increase in luciferase refolding by Hsp90 is lower in presence of the mutant Hsp70s than the wt isoform. Thus collectively, these observations suggest that Hsp90 is relatively less efficient with mutant Ssa1 Hsp70s indicating a potential role of the Hsp70 subregion encompassing these mutations in Hsp90 functions.

The loss of v-Src maturation leads to its aggregation and subsequent degradation [60]. The reduced phosphorylation of cellular proteins in A1-T175N and A1-D158N expressing v-Src suggests that the kinase activity is adversely affected in these strains. In agreement with lower kinase activity, v-Src was primarily found in the pellet fraction of cellular lysate indicating that the kinase is not natively folded. Interestingly there is no enhanced degradation of the misfolded client in strains expressing Ssa1 mutants which resulted in accumulation of intracellular v-Src aggregates, as observed in cell-pellet fraction. Why misfolded v-Src or also other kinase Ste11ΔN is not degraded remains to be studied however it could be due to altered interaction of the mutant Hsp70s/Hsp90s with its co-chaperones required for their degradation. Indeed, the mutant Hsp70s binds with relatively higher affinities with Ydj1, which in turn could affect their interaction with other co-chaperones such as Sse1 and Fes1, known to facilitate Hsp70 degradation activity [61,62].

Hsp40s not only stimulate Hsp70 activity but also regulates its functional specificity [63]. The pull-down with His_6_-Ydj1 as bait protein shows that the Ydj1 binds with higher affinity to mutant Ssa1s than with wt Ssa1. Also, as compared to wt Ssa1, the Ssa1-T175N and Ssa1-D158N are 1.5 to 2 fold more active in refolding denatured luciferase indicating that increased affinity of Ydj1 with the mutant Hsp70s further enhances their substrate refolding activity. The luciferase refolding efficiency was further increased upon addition of Hsp90 however, interestingly, the enhancement was found to be higher in reaction containing wt Ssa1 than the mutants suggesting that the mutants cooperate relatively poorly with Hsp90 in substrate refolding. The in vitro results are also in agreement with in vivo findings showing lower client maturation in cells expressing the Hsp70 mutants than wt Ssa1 as a sole source of Ssa Hsp70. As the C-terminal domain of wt and the mutant Ssa1 Hsp70s is identical, the data suggest that the ability of Hsp90 in substrate refolding is not only dependent upon its interaction through Hsp70-C terminus but also on its interaction with the nucleotide-binding domain of Hsp70.

The Hsp90 interacts with nucleotide-binding as well as C-terminal domains of Hsp70s. The in vitro BLI analysis confirms that the direct Hsp70/Hsp90 interaction is weaker with Ssa1 mutants. The in vivo pull-down analysis for Ssa1 interacting partners showed a lower abundance of the Hsp70/Hsp90 complex in A1-T175N and A1-D158N strains, even though Sti1 is expressed in the cells. The data indicate that Hsp90 binding to the nucleotide-binding domain of Hsp70 might regulate downstream interaction of Hsp90-Sti1 with C-terminal domain of Hsp70. Since Ssa1 mutants interact with higher affinity to Ydj1, it is likely that the co-chaperone interaction at NBD leads to conformational changes in Hsp70 affecting its interaction with the bridge protein Sti1. This is in agreement with previous findings that the co-chaperone interaction at the nucleotide binding domain of Hsp70 affects conformational changes in the substrate binding and the C-terminal domain of the protein [64]. The increased affinity of Ydj1 with the mutant Ssa Hsp70s would make it less likely to be free for further interaction with the Sti1-Hsp90 resulting in a reduced level of the ternary complex. The relatively lower increase in luciferase refolding upon addition of Sti1-Hsp90 to refolding reaction containing mutant Ssa1 is likely to be related to weak interaction of the mutant Ssa Hsp70 with wt Hsp90. Overall, these results suggest that the nucleotide-binding domain of the Hsp70 regulates its C-terminal mediated interaction with Sti1-Hsp90.

The pull-down assay with Ydj1 as a bait protein showed that it binds with similar affinity to Hsp90 in strains expressing Ssa1-T175N or Ssa1-D158N. This interaction of Ydj1 with Hsp90 is more likely to be a direct interaction rather than through Hsp70 as Ydj1 binds with different affinities to mutant Ssa1 Hsp70 though interaction with Hsp90 is similar in strain expressing different Ssa1 mutants. As Ydj1 interaction with Hsp90 remains similar in cells expressing wt or the mutant Hsp70, the Ydj1-Hsp90 direct collaboration is less likely to be the basis of either reduced v-Src maturation or lower Hsp70-Hsp90 complex formation in A1-T175N or A1-D158N strain.

The Sti1 and not other Hsp90 co-chaperones, was found to be essential for cellular growth in strain expressing Ssa1-T175N or Ssa1-D158N as sole Ssa Hsp70, suggesting that it’s primarily the client transfer activity from the Hsp70 to Hsp90 that is impaired in these strains. The growth defect was more pronounced at a higher temperature even in the presence of Sti1. Since at higher temperature proteins are more prone to misfold and aggregate, the cellular demand for a functional Hsp70 and Hsp90 chaperones would increase with an increase in temperature. The reduced cellular growth of A1-T175N and A1-D158N could be due to the compromised Hsp70 activity. Alternatively, as the Hsp70 mutations lie in an interacting region of Hsp90 [16,59], it’s possible that in these strains Hsp90 activity is compromised in a manner that is not compensated by the interaction of Hsp70-Hsp90 by Sti1. This suggests non-redundant roles of Hsp70-Hps90 interaction through the region surrounding amino acids T175/D158 versus bridge protein Sti1. The Ydj1 is synthetically lethal with Sti1 as the *ydj1Δsti1Δ* remains inviable [65]. It is interesting to note that Ydj1 interacts better with Ssa1 mutants (Ssa1-T175N and Ssa1-D158N) and also promote their ability to refold denatured luciferase suggesting that the growth defect upon Sti1 deletion in these mutants is not due to depletion of Ydj1 function.

Among the various Sti1 derivatives that were examined, none except that lacking linker region could complement Sti1 function in supporting cellular growth in A1-T175N and A1-D158N suggesting that the linker region is not essential for such Sti1 function. Interestingly, Sti1 derivative lacking only DP2 region also did not support growth suggesting an important role of the domain in client protein maturation. This is in agreement with a previous study suggesting the essential role of DP2 in client activation, possibly by either direct binding to substrates or facilitating their conformational changes required for client transfer to Hsp90 [12]. DP2 alone also could not complement the Sti1 function. Further, as construct lacking DP2 contains all Sti1 region important for Hsp70 and Hsp90 interaction yet not able to complement Sti1 function suggest that the bridging the two chaperones alone is not sufficient for Hsp90 chaperoning activity in A1-T175N and A1-D158N strains. In summary, since Sti1 does not have a client folding activity of its own, and yet is required to support growth in A1-T175N and A1-D158N suggests that the client transfer activity from Hsp70 to Hsp90 gets adversely affected in cells expressing Ssa1-T175N or Ssa1-D158N as sole Ssa Hsp70.

**Fig 11:**
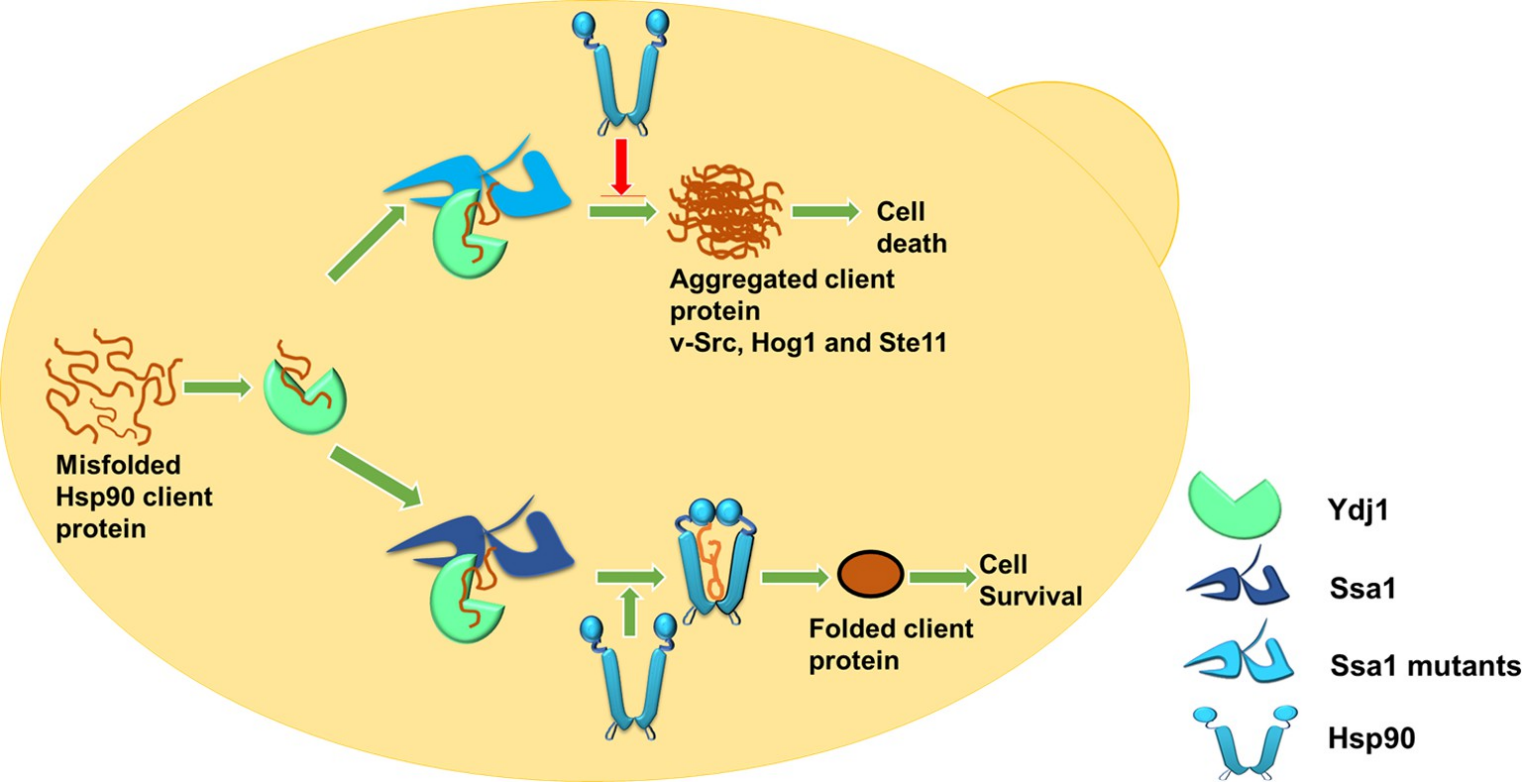
Model showing regulation Hsp90 binding to Hsp70 by Ydj1. Misfolded Hsp90 client protein interacts with Ydj1. Ydj1 delivers client proteins to Hsp70. Absence of Hsp90 interaction with Hsp70 mutants due to their higher affinity with Ydj1 results in client aggregation. Red arrow represents lack of Hsp90 interaction with Ssa1 mutants.

The modeled structure of the complex of nucleotide-binding domain of Ssa1 with the J-domain of Ydj1 shows that the identified mutation is in the interacting region of the two proteins. Indeed, our pull-down study with His_6_-Ydj1 as bait protein as well as BLI study show that the Ssa1 mutants bind with higher affinity to Ydj1. Further, T175 and D158 lie in the Hsp70 region that interacts with Hsp90 [16,59]. The identified T175N mutation is homologous to the mutation found in DnaK that affects its binding with Hsp90. Overall, these findings suggest that the identified Ssa1 mutations are at a region that interacts with both Ydj1 and Hsp90. As the Ssa1 mutants bind with lower affinity to Hsp90, it’s possible that under in vivo conditions, both Hsp90 and Ydj1 compete for the same region in Ssa1, and Ydj1 having relatively higher affinity to the mutant Hsp70s competes out Hsp90. Further decrease in Hsp90 binding to Hsp70 in A1-T175/D158 lacking Sti1 might result in complete disruption of formation of Hsp70-Hsp90 complex resulting in the cellular lethality (Fig 11). This is in agreement with the data showing that mutation homologous to Ssa1-T175N or Ssa1-D158N in Ssa4, can support cellular viability in the absence of Sti1. As Ydj1 interacts with a lower affinity with Ssa4 than Ssa1, it might not be able to compete out Hsp90 for its binding with Ssa4 mutants [26]. The current study show the effect of identified mutation on Hsp90 substrates v-Src, Ste11 and Hog1, and similar effect on other substrates remains to be seen.

The reduced growth defect in *sti1Δ* cells with D158N as compared to T175N mutant of Ssa2/3 could be related to their difference in the binding affinity to Ydj1. Indeed the binding affinity of Ssa1-T175N is relatively higher than Ssa1-D158N with Ydj1. The growth defect of T175N was found to be more pronounced with Ssa3, as cells expressing Ssa3-T175N grew much poorly even in the presence of Sti1. The results further supports previous findings that though highly homologous, the different Ssa Hsp70 chaperones function distinctly in Hsp90 chaperone pathway [26]. Overall these results show that Ydj1 affinity to Ssa Hsp70s plays a critical role in formation of Hsp70-Hsp90 complex and thus Hsp70 role in Hsp90 chaperoning pathway. The role of other Hsp40s that interact to a similar region of Hsp70, in the Hsp90 chaperoning function remains to be determined. Also, the current study is carried out with strains expressing desired Ssa Hsp70 in the absence of other three isoforms, and thus how the presence of different Hsp70 isoforms affect interactions and thus coordination with their co-chaperones in wt strain need to be further explored.

Recent studies show the significance of postranslational modifications of chaperones in their cellular functions, also known as chaperone code [66]. Interestingly, T175 in Ssa1 is known to be phosphorylated, and D158 has proximity to sites on Ssa1 that undergo modifications [67]. Thus its possible that alteration of these modifications by substitution of residues at 158 and 175 to asn affects the chaperone function resulting in loss of Hsp90 client maturation as well as growth defect upon deletion of Sti1.

Both Hsp70s and Hsp90s play crucial role in the maintenance of a wide range of cellular proteins. Thus cellular health is quite susceptible to changes in either Hsp70 or Hsp90 activity. In prokaryotes, that lack bridge protein Sti1, the sole mode of Hsp70-Hsp90 interaction is through the nucleotide-binding domain of Hsp70. Though the Hsp90 interaction with Hsp70 nucleotide-binding domain remains conserved in eukaryotes, they evolved an additional mode of interaction through bridge protein Sti1. We show that the Hsp90 interaction at the NBD of Hsp70 has a dominant effect on its interaction with the C-terminal domain of Hsp70 through Sti1. The bimodal interaction of Hsp90 with Hsp70 provides an additional regulation in determining the functional specificities within highly homologous Hsp70s beneficial for better fitness of cells under stressful conditions. It is possible that under optimal conditions with lower load of misfolded proteins, the requirement of Hsp90s is low. Under these conditions, more of Ydj1 is involved in Hsp70 activation required for many diverse cellular functions. Under stress conditions, the Ydj1 might get sequestered with a much larger fraction of misfolded proteins, resulting in higher interaction of Hsp90 with NBD domain of Hsp70 and thus increased abundance of Hsp70-Hsp90 complex required for maintenance of protein homeostasis.

## Materials and methods

### Strains and plasmids

The strains and plasmids used in this study are described in Tables 4 and 5.

**Table 4:** Srains used in the present study.

Strain	Genotype	Reference
**SY187**	*MATa*, *kar 1–1*,*P*_*DAL5*_::*ADE2*, *his3Δ202*, *leu2Δ1*, *trp1Δ63*, *ura3-52*	[70]
**SY135**	*MATa*,*P*_*DAL5*_::*ADE2*, *ssa1*::*Kan*, *ssa2*::*HIS3*, *ssa3*::*TRP1*, *ssa4*::*ura3-2f/pRS315P*_*SSA2*_*-SSA1*	[78]
**SY136**	*MATa*, *P*_*DAL5*_::*ADE2*, *ssa1*::*Kan*, *ssa2*::*HIS3*, *ssa3*::*TRP1*, *ssa4*:: *ura3-2f/pRS315P*_*SSA2*_*-SSA2*	[78]
**SY143**	*MATa*, *P*_*DAL5*_::*ADE2*, *ssa1*::*Kan*, *ssa2*::*HIS3*, *ssa3*::*TRP1*, *ssa4*:: *ura3-2f/pRS315P*_*SSA2*_*-SSA3*	[78]
**SY211**	*MATa*, *P*_*DAL5*_::*ADE2*, *ssa1*::*Kan*, *ssa2*::*HIS3*, *ssa3*::*TRP1*, *ssa4*:: *ura3-2f/pRS315P*_*SSA2*_*-SSA4*	[78]
**DD179**	*MATa*, *P*_*DAL5*_::*ADE2*, *ssa1*::*Kan*, *ssa2*::*HIS3*, *ssa3*::*TRP1*, *ssa4*:: *ura3-2f/pRS315P*_*SSA2*_*-SSA1-A371T D577N*	This study
**DD180**	*MATa*, *P*_*DAL5*_::*ADE2*, *ssa1*::*Kan*, *ssa2*::*HIS3*, *ssa3*::*TRP1*, *ssa4*:: *ura3-2f/pRS315P*_*SSA2*_*-SSA1-Δ604–642*	This study
**DD201**	*MATa*,*P*_*DAL5*_::*ADE2*, *ssa1*::*Kan*, *ssa2*::*HIS3*, *ssa3*::*TRP1*, *ssa4*::*ura3-2f/pRS315P*_*SSA2*_*-SSA1- T175N*	This study
**DD202**	*MATa*,*P*_*DAL5*_::*ADE2*, *ssa1*::*Kan*, *ssa2*::*HIS3*, *ssa3*::*TRP1*, *ssa4*::*ura3-2f/pRS315P*_*SSA2*_*-SSA1- A464V*	This study
**DD203**	*MATa*,*P*_*DAL5*_::*ADE2*, *ssa1*::*Kan*, *ssa2*::*HIS3*, *ssa3*::*TRP1*, *ssa4*::*ura3-2f/pRS315P*_*SSA2*_*-SSA1- D158N*	This study
**DD204**	*MATa*,*P*_*DAL5*_::*ADE2*, *ssa1*::*Kan*, *ssa2*::*HIS3*, *ssa3*::*TRP1*, *ssa4*::*ura3-2f/pRS315P*_*SSA2*_*-SSA1- A275V*	This study
**DD205**	*MATa*, *P*_*DAL5*_::*ADE2*, *ssa1*::*Kan*, *ssa2*::*HIS3*, *ssa3*::*TRP1*, *ssa4*:: *ura3-2f/pRS416P*_*GPD*_*-His*_*6*_*SSA1*	This study
**DD206**	*MATa*, *P*_*DAL5*_::*ADE2*, *ssa1*::*Kan*, *ssa2*::*HIS3*, *ssa3*::*TRP1*, *ssa4*:: *ura3-2f/pRS416P*_*GPD*_*-His*_*6*_*SSA1-T175N*	This study
**DD207**	*MATa*, *P*_*DAL5*_::*ADE2*, *ssa1*::*Kan*, *ssa2*::*HIS3*, *ssa3*::*TRP1*, *ssa4*:: *ura3-2f/pRS416P*_*GPD*_*-His*_*6*_*SSA1-D158N*	This study
**SY289**	*MATa*, *P*_*DAL5*_::*ADE2*, *ssa1*::*NAT*, *ssa2*::*HIS3*, *ssa3*::*TRP1*, *ssa4*:: *ura3-2f*, *sti1*::*KanMX/pRS316P*_*SSA2*_*-SSA2*	This study
**DD502**	*MATa*, *P*_*DAL5*_::*ADE2*, *ssa1*::*NAT*, *ssa2*::*HIS3*, *ssa3*::*TRP1*, *ssa4*:: *ura3-2f*, *sti1*::*KanMX/ pRS315P*_*SSA2*_*-SSA1/pRS327-STI1*	This study
**NK402**	*MATa*, *P*_*DAL5*_::*ADE2*, *ssa1*::*NAT*, *ssa2*::*HIS3*, *ssa3*::*TRP1*, *ssa4*:: *ura3-2f*, *sti1*::*KanMX/ pRS315P*_*SSA2*_*-SSA1/pCM189-His*_*6*_*-STI1*	This study

**Table 5:** Plasmids used in the present study.

Plasmid	Marker	Reference
pRS316P_GAL1_-FLAG-vSrc	URA3	[26]
pRS316P_GAL1_-His_6_-STE11ΔN	URA3	This study
pRS412P_GAL1_-STE11ΔN	ADE2	This study
PRE-*lacZ*	URA3	[32]
pRS315P_SSA2_-SSA1	LEU2	[78]
pRS315P_SSA2_-SSA2	LEU2	[78]
pRS315P_SSA2_-SSA3	LEU2	[78]
pRS315P_SSA2_-SSA4	LEU2	[78]
pRS315P_SSA2_-SSA1-T175N	LEU2	This study
pRS315P_SSA2_-SSA1-D158N	LEU2	This study
pRS315P_SSA2_-SSA1-Δ6604–642	LEU2	This study
pRS315P_SSA2_-SSA1-A464V	LEU2	This study
pRS315P_SSA2_-SSA1-A371T D577N	LEU2	This study
pRS315P_SSA2_-SSA1-A275V	LEU2	This study
pRS315P_SSA2_-SSA2-T175N	LEU2	This study
pRS315P_SSA2_-SSA2-D158N	LEU2	This study
pRS315P_SSA2_-SSA3-T175N	LEU2	This study
pRS315P_SSA2_-SSA3-D158N	LEU2	This study
pRS315P_SSA2_-SSA4-T175N	LEU2	This study
pRS315P_SSA2_-SSA4-D158N	LEU2	This study
pRS416P_GPD_-His_6_SSA1	URA3	This study
pRS416P_GPD_-His_6_SSA1-T175N	URA3	This study
pRS416P_GPD_-His_6_SSA1-D158N	URA3	This study
pCM189-STI1	URA3	This study
pRS327-GPD-STI1	LYS2	This study
pRS327-GPD-STI1ΔLinker	LYS2	This study
pRS327-GPD-STI1-DP2	LYS2	This study
pRS327-GPD-STI1-TPR-DP1-TPR2A	LYS2	This study
pRS327-GPD-STI1-TPR2B	LYS2	This study
pRS327-GPD-STI1-ΔDP2	LYS2	This study
pRS327-GPD-STI1-TPR2A-TPR2B-DP2	LYS2	This study
pPROEXHTV-YDJ1	Ampicillin,	[70]
pET29bHTV-HSP82	Kanamycin	[26]
pET29bHTV-STI1	Kanamycin	[26]
HSE-*lacZ*	URA3	[79]
pMR135	URA3	[72]
pRS317-P_tetoff_-SSA1	LYS2	This study

Strain SY135 (A1) has pRS315-SSA1 as sole source of Ssa Hsp70 isoform. The gene encoding Sti1, Hsp82, Hsc82, Cpr6, Cpr7, Sba1, Aha1, Hch1, Tah1 and Pih1 was knocked out using standard homologous-based recombination approach.

For protein purification, the gene encoding Ssa1 was PCR amplified, digested with BamHI and XhoI, and subcloned into plasmid pRS416-P_GPD_-His_6_ digested with same enzymes to generate pRS416-P_GPD_-His_6_SSA1. The plasmid encode from 5’ to 3’ direction His_6_-tag, TEV protease site and gene encoding Ssa1. Similarly, Ssa1-T175N and Ssa1-D158N were subcloned to generate plasmids pRS416-P_GPD_- His_6_SSA1-T175N and pRS416-P_GPD_- His_6_SSA1-D158N respectively.

PCR amplified His_6_-STI1 was cloned in pCM189 vector at BamH1 and Xma1 Sites. *STI1* under native promoter and terminator was cloned in pRS327 vector using Pst1 and HindIII restriction sites. All constructs were further confirmed by DNA sequencing.

### Random mutagenesis

pRS315-SSA1 was mutagenized using Forsburg hydroxylamine mutagenesis−based method [68]. The mutagenesis buffer is composed of 0.35 g hydroxylamine hydrochloride, 450 μL of 5 M NaOH, 4.55 mL of ice-cold sterile MQ water, pH 6.7. 10 μg of pRS315-SSA1 was incubated in 500 μl of mutagenesis buffer for 48 hours. The mutant library thus obtained was purified using GeneJET PCR purification kit (ThermoFisher, catalog number 0702) and used for further transformation in *S*. *cerevisiae*.

### Media and growth conditions

Media composition is similar to as described before [69].

For induction by galactose driven promoter, cells were grown in liquid SRaff growth media until 1 O.D._600nm_. The cells were collected by centrifugation, washed with sterile water and further subcultured at 0.5 O.D._600nm_ for 6 hours in SGal media. The cell cultures were grown at 30°C unless otherwise mentioned.

### Immunoblot analysis

Cells grown in liquid media were harvested by centrifugation, and lysed using glass beads. The lysis was carried out using bead ruptor (OMINI BEAD RUPTOR 24) at 3000 rpm for 30 seconds. The lysate was further fractionated into supernatant and pellet. 10–20 μg of total protein was separated onto 10% SDS-PAGE and transferred onto PVDF membranes. The primary antibodies used in the study are as follows: anti-FLAG antibody (F3165 Sigma), anti-Phosphotyrosine (05–321 Millipore), anti-Hsc82 (ab30920 Abcam), anti-Ydj1 (SAB5200007 Sigma), anti-His_6_ antibodies (Pierce, USA-MA1-21315), anti-Hsp70 (ADI-SPA-822-F Enzo Lifesciences), and anti-Pgk1 (catalog number 459250 Invitrogen). Secondary antibodies used in this study include anti-mouse IgG HRP linked (7076, Cell signalling technologies) and anti-rabbit IgG HRP (7074, Cell signalling technologies).

### Protein purification

Hsp82 and Sti1 were purified as described earlier [26]. Ydj1 was purified similar to method mentioned before [70].

Ssa1, Ssa1-T175N and Ssa1-D158N were purified as described earlier [69]. Strain harboring plasmid pRS416-P_GPD_-His_6_SSA1, pRS416-P_GPD_-His_6_SSA1-T175N or pRS416-P_GPD_-His_6_SSA1-D158N was grown in liquid YPAD media for 24–48 hours at 30°C. Cells were centrifuged and resuspended into buffer containing 20 mM HEPES, 150 mM NaCl, 20 mM KCl and 20 mM MgCl_2_, pH 7.4. Cells were lysed using glass beads followed by sonication for 30 minutes. Nickel based metal affinity resin was used for the purification of Hsp70s. The His_6_ tag was removed by incubation of purified protein with His_6_-TEV. The reaction mixture was incubated with cobalt based metal affinity resin, and TEV protease was removed in the bound fraction. Protein purity was confirmed onto 10% SDS-PAGE.

### Immunoprecipitaion and pull down

Immunoprecipitation was performed as described earlier [26]. Briefly, cells were resuspended in 20 mM Tris (pH 7.5) buffer containing 150 mM NaCl, 0.5 mM EDTA, 0.1% Triton-X100, 1 mM PMSF, and lysed using glass beads (OMINI BEAD RUPTOR 24). The clarified lysate was incubated with anti-FLAG antibody conjugated agarose resin (Sigma A2220) for 16h at 4°C. The beads were washed with buffer containing 150 mM NaCl, 0.1% Triton X-100 in 20 mM Tris (pH 7.5). Immunoprecipated proteins were separated onto 10% SDS-PAGE, transferred onto PVDF membrane and probed with appropriate antibodies

The pull-down assay using purified proteins was performed as described earlier [71]. Purified His_6_-Ydj1 was bound to cobalt based affinity resin for 1 hour, followed by incubation with clarified yeast lysate for 1h. Bound fraction was eluted with buffer containing 20mM EDTA.

The His_6_-Ssa1, His_6_-Ssa1-T175N and His_6_-Ssa1-D158N expressed from plasmid borne gene were used for in vivo pull-down studies. Briefly, clarified yeast lysate was incubated with cobalt based metal affinity resin for 1h. The beads were further washed with buffer containing 20 mM HEPES, 150 mM NaCl, 20 mM KCl and 20 mM MgCl_2_, 60 mM Imidazole and 0.1% Tween-20, pH 7.4. Bound proteins were eluted using buffer containing 20 mM HEPES, 500 mM NaCl, 20 mM KCl and 20 mM MgCl_2_ and 300 mM Imidazole.

### Quantitative real-time PCR

Quantitative Real-Time PCR was used to study mRNA transcripts of *v-SRC*. 18S-rRNA was monitored as housekeeping gene. The cells were harvested and total RNA was purified using HiPurA Yeast RNA Purification Kit (HiMedia, MB611). About 100ng of RNA was used to prepare cDNA using iScript Select cDNA Synthesis Kit (Bio-rad, 1708896). 50 ng of cDNA was used as template for quantitative Real-Time PCR (qRT-PCR) using Biorad SYBR kit (1725270) on Biorad Real-time PCR system.

### Luciferase refolding assay

Luciferase refolding was performed as described earlier [26]. Firefly luciferase (80 nM) was denatured by incubating it at 45°C for 7 minutes. The refolding of denatured luciferase (40 nM) was carried out in the presence of 0.6 μM Ydj1 and 2 μM of Hsp70 (Ssa1, Ssa1-T175N or Ssa1-D158N). Refolding was performed at 25°C and initiated upon addition of 1 mM ATP. Effect of Hsp90 on luciferase refolding was investigated by incubating denatured luciferase (40 mM) in the presence of 0.3 μM Ydj1, 0.5 μM of Hsp70 (Ssa1, Ssa1-T175N or Ssa1-D158N), 2.4 μM Sti1 and 0.9 μM Hsp82.

### In vivo luciferase refolding

Cells harboring pMR135 were grown in liquid SD media until O.D._600nm_ reaches ~1. Cells were harvested and resuspended in fresh liquid SD media to 0.3 O.D._600nm_. 100 μg/ml of cycloheximide was added to cells suspension to stop protein synthesis. To denature luciferase, cells were incubated at 48°C for 30 minutes. Luciferase was further refolded by shifting cell to 30°C. The refolding was monitored by adding 50μl of D-luciferin (23 μg/sample) to 200μl of cell aliquot [72].

### Ste11 kinase assay

Cells were co-transformed with pRS412-P_GAL1_-Ste11ΔN and PRE-*lacZ* or only with PRE-*lacZ* [32]. Transformants with pRS412-P_GAL1_- His_6_ Ste11ΔN and PRE-*lacZ* were grown in liquid SD media until 1–1.2 O.D._600nm_ and further subcultured at 0.5 O.D._600nm_ in SGal growth media to induce His_6_-Ste11ΔN expression for 6 hours. Transformants with PRE-*lacZ* alone were grown in liquid SD media until 1–1.2 O.D._600nm_ and further subcultured at 1 O.D._600nm_ in SD media containing 5 μM of α-factor for 6 hours. To monitor β-galactosidase activity, 1 O.D._600nm_ cells were permeabilized using 3 freeze thaw cycle in liquid N_2_ and further incubated with 200 μl of ortho-Nitrophenyl-β-galactoside (ONPG (4 mg/ml)) at 30°C for 15 minutes followed by addition of 200 μL of 1M Na_2_CO_3_. The culture was centrifuged at 13000 rpm for 10 minutes and supernatant was used for measuring absorbance at 420nm.

### HSE-*lacZ* assay

Cells were transformed with HSE-*lacZ* [73]. Transformants were grown until 1–1.2 O.D._600nm_ at 30°C. Cells were further normalized at 1 O.D._600nm_ and incubated at 37°C for 4 hours. The β-galactosidase activity was monitored as described above for PRE-*lacZ* assay.

### CD analysis

Far UV CD spectra were recorded on a JASCO-J-815 spectropolarimeter. The CD spectra were recorded over 250–195 nm in a cuvette of 1-mm pathlength with 10 nm/min of scan rate at 25°C. The CD signal was converted into Mean Residue ellipticity (MRE) as described before [74,75].

### Bio-layer intereferometry

Bio-layer interferometry studies were carried out in Octet K2 instrument (Fortebio) at 30°C. Hsp90 was immobilized on Amine Reactive 2^nd^ Generation (AR2G) biosensors by amine coupling chemistry using 1:1 ratio of 0.1 M N-Hydroxysuccinimide (NHS) and 0.4 M 1-ethyl-3-(3-dimethylaminopropyl)-carbodiimide (EDC). Biosensor treated as above except with buffer lacking Hsp90 was used as reference control. The biosensors were then dipped into 1M ethanolamine solution for 600 seconds. Ssa1 and its mutants were prepared in 1X assay buffer (25 mM Hepes (pH 7.4), 150 mM NaCl, 20mM MgCl_2_, 20mM KCl) containing 0.1% Tween-20 (v/v) and 0.05% Triton-X-100 (v/v) along with 1 mM ATP. Association was monitored for 200 seconds by dipping the Hsp90 bound biosensors into various concentrations of Ssa1 or its mutants. The dissociation was monitored by dipping the biosensors in 1X assay buffer at shaking speed of 1000 rpm for 200 seconds. The non-specific signal obtained from reference biosensor was subtracted from corresponding signal obtained upon Hsp90 immobilized biosensors. Binding curves was further analyzed using software ‘Data Analysis 9.0’ available from ForteBio.

BLI for Ydj1 and Hsp70 was performed as described earlier [26].

### Modelling

The 3D structure of Hsp70 from *Saccharomyces cerevisiae* was taken from AlphaFold Protein Structure Database (https://alphafold.ebi.ac.uk/entry/P10591). Hsp70 NBD region was extracted from the 3D structure and docked with Hsp40 J-domain (pdb id 5VSO) from *Saccharomyces cerevisiae* on HDOCK server. The docked complex was visualised in Discovery Studio to identify the interacting residues.

### Yeast cell lysate fractionation and sample preparation for TMT based QMS analysis

Tet-OFF promoter regulatable Sti1 plasmid harboring DD502 and NK402 strains were grown into dextrose synthetic medium without uracil amino acid until saturation. 0.025 O.D._600nm_ cells were inoculated in secondary culture and grown until log phase. Cells were pelleted, washed with 1X PBS and 40 O.D._600nm_ cells were collected for yeast cell lysate fractionation (0h time point). 0.06 O.D._600nm_ cells were inoculated in 10 μg/ml of Dox containing repression medium and grown for 10 hr. After 10 hr cells were pelleted, washed and 40 O.D._600nm_ cells were collected for yeast cell lysate fractionation (10h time point). 40 O.D._600nm_ cells were lysed in 1 ml of lysis buffer (25 mM HEPES, 150 mM NaCl, 1 mM EDTA, 1 mM EGTA, 10% Glycerol) using zirconium beads in Fast Prep Zymo bead beator at 4000 rpm of 30 on/ 30 off for 4 cycles at 4°C. Cell debris were removed by spinning at 4000 rpm for 1 min. Clarified lysates were spun for 13000 rpm for 30 min and fractionated into supernatant and pellet fractions. 30 μg of total proteins in supernatant fractions were used for TMT based QMS analysis.

### Tryptic digestion and Tandem Mass Tag (TMT) labelling

Samples were reduced with 100 mM DL-dithiothreitol (DTT) at 56°C for 30 min and processed using the modified filter-aided sample preparation (FASP) method [76]. In short, reduced samples were transferred to 30 kDa MWCO Pall Nanosep centrifugation filters (Pall Corporation) and washed twice with 8 M urea. Additional washes with digestion buffer (0.5% sodium deoxycholate in 50 mM TEAB) was performed before and after alkylation with 10 mM methyl methanethiosulfonate for 20 min at room temperature. Protein digestions were performed using Trypsin (Pierce MS grade) in digestion buffer, first with 0.3 μg Trypsin at 37°C overnight followed by new addition of 0.3 μg trypsin and incubation at 37°C for three hours. Produced tryptic peptides were collected by centrifugation and labelled using TMT 10-plex isobaric mass tagging reagents (Thermo Scientific) according to the manufacturer instructions. Labelled samples were combined and sodium deoxycholate was removed by acidification with 10% TFA. Peptides were desalted using Pierce Peptide Desalting Spin Columns (Thermo Scientific) following the manufacturer’s instructions.

The combined desalted TMT-labeled sample was fractionated by basic reversed-phase chromatography (bRP-LC) using a Dionex Ultimate 3000 UPLC system (Thermo Fischer Scientific). Peptide separations were performed using a reversed-phase XBridge BEH C18 column (3.5 μm, 3.0x150 mm, Waters Corporation) and a gradient from 3% to 100% acetonitrile in 10 mM ammonium formate buffer at pH 10.00 over 22 min. The fractions concatenated into 15 or 22 fractions that were evaporated and reconstituted in 20 μl of 3% acetonitrile, 0.2% formic acid for nLC-MS/MS analysis.

### nLC-MS/MS

The fractions were analyzed on an orbitrap Lumos or Fusion Tribrid mass spectrometer interfaced with Easy-nLC1200 liquid chromatography system (Thermo Fisher Scientific). Peptides were trapped on an Acclaim Pepmap 100 C18 trap column (100 μm x 2 cm, particle size 5 μm, Thermo Fischer Scientific) and separated on an in-house packed analytical column (75 μm x 35 cm, particle size 3 μm, Reprosil-Pur C18, Dr. Maisch) using a gradient from 3% to 100% acetonitrile in 0.2% formic acid over 85 min at a flow of 300 nL/min. MS scans were performed at 120 000 resolution. MS/MS analysis was performed in a data-dependent, with top speed cycle of 3 s for the most intense doubly or multiply charged precursor ions. Precursor ions were isolated in the quadrupole with a 0.7 m/z isolation window, with dynamic exclusion set to 10 ppm and duration of 45 seconds. Isolated precursor ions were subjected to collision induced dissociation (CID) at 35 collision energy. Produced MS2 fragment ions were detected in the ion trap followed by multinotch (simultaneous) isolation of the top 10 most abundant fragment ions for further fragmentation (MS3) by higher-energy collision dissociation (HCD) at 65% and detection in the Orbitrap at 50000 resolutions, m/z range 100–500.

### Proteomic data analysis

Identification and relative quantification was performed using Proteome Discoverer version 2.4 (Thermo Fisher Scientific). The Swissprot Saccharomyces cerevisiae proteome database (December 2019) was used for the database search, using the Mascot search engine v. 2.5.1 (Matrix Science, London, UK) with MS peptide tolerance of 5 ppm and fragment ion tolerance of 0.6 Da. Tryptic peptides were accepted with 0 missed cleavage; methionine oxidation was set as a variable modification, cysteine methylthiolation, TMT-6 on lysine and peptide N-termini were set as fixed modifications. Percolator was used for PSM validation with the strict FDR threshold of 1%. TMT reporter ions were identified in the MS3 HCD spectra with 3 mmu mass tolerance, and the TMT reporter intensity values for each sample were normalized on the total peptide amount. Only the unique identified peptides were taken into account for the relative quantification. The mass spectrometry proteomics data have been deposited to the ProteomeXchange Consortium via the PRIDE partner repository with the dataset identifier PXD034983 [77].

## Supporting information

S1 Fig Screening of Hsp70 mutants against v-Src mediated toxicity. **(A)** Strain SY244/pRS317-P_Tetoff_-SSA1 was transformed with pRS316 or pRS316-P_GAL1_-vSrc along with either wt-SSA1 or SSA1 mutagenized with hydroxylamine. Cells were plated onto doxycycline containing SD or SGal plates to shut expression of SSA1 under Tet repressible promoter. Shown is growth after 5 days of incubation at 30°C. **(B)** Conservation of the residues near identified Hsp70 mutants in Hsp70 isoforms from *S*. *cerevisiae* (Ssa1-Ssa4) and Human (Hsp70 and Hsc70).
(TIF)

S2 Fig Strains expressing Ssa1-T175N and Ssa1-D158N show reduction in v-Src toxicity. *S*. *cerevisiae* strains A1, A1-T175N and A1-D158N were transformed with (EV) or pRS316P_GAL1_-FLAG-v-Src (FLAG-v-Src). Transformants were pooled and grown into selective SD media. Cells were washed and serially diluted onto SD and SGal media. Shown is growth after 5 days of incubation at 30°C.
(TIF)

S3 Fig Ssa1-T175N or Ssa1-D158N effect on v-Src toxicity is recessive. *S*. *cerevisiae* strain SY187 was transformed with pRS316 (EV) or pRS316P_GAL_-FLAG-v-Src (FLAG-v-Src) and pRS315-SSA1 or pRS315-SSA1* (T175N or D158N). Transformants were grown in selective liquid SD media overnight. Cells were further serially diluted and spotted onto SD and SGal media. Shown here is the growth after 4 days of incubation at 30°C.
(TIF)

S4 Fig Major chaperones are expressed similarly in A1, A1-T175N and A1-D158N strains. Yeast lysate was prepared from indicated strains grown in inducible liquid SGal media for 6 hours. 5μg (1X) or 10μg (2X) of total lysate protein was loaded into each lane and probed with anti-Ydj1, anti-Sse1, anti-Hsp104, and anti-Pgk1 antibodies.
(TIF)

S5 Fig CD spectra for Ssa1, Ssa1-T175N and Ssa1-D158N. Far-ultraviolet circular dichroism spectroscopic analysis of wt-Ssa1, Ssa1-T175N and Ssa1-D158N. The 10 μM protein in 2.5 mM HEPES buffer containing 15 mM NaCl, pH 7.5 in a 1-mm path length cuvette was used to record CD spectra.
(TIF)

S6 Fig Strains A1-T175N and A1-D158N show reduced Ste11 pathway activity. **(A)** Cells harboring Ste11ΔN encoding plasmid were grown into selective liquid SD media, and further serially diluted onto solid SD and SGal media. Shown is growth after 4 days of incubation at 30°C. **(B)** Immunoblot showing His_6_-Ste11ΔN steady-state level in indicated strains. The anti-His_6_ was used as the primary antibody. **(C)** Indicated strains were co-transformed with plasmid overexpressing Ste11ΔN and pPRE-*lacZ*. Ste11ΔN was induced by growing in SGal media. β-galactosidase activity was measured similar to as described above for Ste11. **(D)** Indicated strains harboring plasmid pPRE-*lacZ* were grown in SD liquid media. Shown is the β-galactosidase activity measured with similar number of cells harvested during mid-log phase. The activity was calculated in Miller units. Error bars represent standard error from 3 different biological replicates.
(TIF)

S7 Fig Alignment of amino-acids sequence of nucleotide-binding domains of Ssa1 with DnaK from *E*. *coli* using the Needleman-Wunsch algorithm for global alignment. (TIF)

S8 Fig (A) Superposed 3D structures of docked NBD(Hsp70)-J-domain (Hsp40) complex from S.cerevisiae (magenta) and from E.coli (blue). Structure for E.coli Hsp70 and Hsp40 are from PDB ID 5NRO. The RMSD of this alignment is 0.475 Å. (B) the relevant residues are highlighted in modelled complex of S. cerevisiae NBD of Ssa1 (blue) and J domain of Ydj1 (red). Q373, L377, E215, I213, E210 and R169 in green are interacting residues of NBD and H34, P35 and D36 in magenta is HPD motif of J-domain. Residues D158 and T175 are also shown in yellow.
(TIF)

S9 Fig Sti1 interacts identically with Ssa1 and Ssa1-T175N. Purified His_6_-Sti1 was incubated with yeast lysate harbouring Ssa1 and Ssa1 T175N as sole source of cytosolic Hsp70. Bound His_6_-Sti1 was further incubated with Co^2+^-NTA resin. Precipitated proteins were immunoblotted with antibodies against Hsp70, Hsp90 and His_6_.
(TIF)

S10 Fig A1-T175N shows a temperature-sensitive growth phenotype. **(A)** A1, A1-T175N or A1-D158N strains were serially diluted and spotted onto YPAD plates. The plates were incubated at 16°C for 5 days, 30°C, and 37°C for 2 days. **(B and C)** *S*. *cerevisiae* strains were grown at 30°C and 37°C respectively. Shown here is O.D._600nm_ at indicated time points. **(D)** The indicated *S*. *cerevisiae* strains harboring plasmid encoding *HSE*-*lacZ* was grown in liquid growth media at 30°C. Subsequently, cells were shifted for heat shock at 37°C for 4 hours. Cells count was normalized and further assayed for β-galactosidase activity as described in material and methods. **(E)** *S*. *cerevisiae* strains expressing thermolabile firefly luciferase were grown at 30°C. Cells were shifted to 48°C for 30min. The refolding was initiated by further incubating cells at 30°C. Shown is the fraction refolded at 30°C in 60 min as compared to that before shifting cells to 48°C. Error bars represent standard error from 3 different biological replicates.
(TIF)

S11 Fig Sti1 linker region is dispensable for its activity required for A1-T175N and A1-D158N strain growth. **(A)** Schematic of domain arrangement of Sti1 and its variants used in the study. **(B)** The *sti1Δ* strains expressing Ssa2 (on Ura3 based plasmid), Ssa1 or its variants (on Leu2 based plasmid), and Sti1 (on Lys2 based plasmid) or its variants were grown onto SD plates and further patched onto FOA plates. The individual Sti1 variant is represented by the same color-code.
(TIF)

S12 Fig The reduction of preformed His_6_-Sti1 abundance in A1 and A1-T175N strains. **(A)** His_6_-Sti1 was expressed from TET repressible promoter in A1 and A1-T175N strains. The doxycycline (repressor) was added in the growth media and His_6_-Sti1 abundance was monitored at indicated time intervals. **(B)** Graph showing the percentage repression of His_6_-Sti1 at different time points with respect to 0 hr in A1 and A1-T175N strains.
(TIF)

S13 Fig The fold increase in luciferase activity upon incubation of Hsp90 with Ssa1-T175N, Ssa1-D158N or wt Ssa1. **(A) and (B)** The bar graph representation of the study shown in Fig 9A and 9B respectively at 30min. Briefly, the denatured luciferase (40nM) was incubated in the presence of 0.3μM Ydj1, 0.5μM Hsp70 (Ssa1, Ssa1-T175N or Ssa1-D158N), 2.5μM Sti1 and 0.9μM Hsp82. Shown here is fold change in luminescence with respect to denatured luciferase after 30 minutes. The bar graph shows the average of the 2 replicates along with the data points. Controls are same as for S13A and S13B Fig.
(TIF)

S1 Excel sheet TMT-based Mass spectrometry proteomic data for A1-T175N strain upon Sti1 repression in soluble fractions. (XLSX)

S2 Excel sheet TMT-based Mass spectrometry proteomic data for A1 strain upon Sti1 repression in soluble fractions. (XLSX)

S1 Dataset Excel file containing numerical data for Fig 1B. (XLSX)

S2 Dataset Excel file containing numerical data for Fig 2A. (XLSX)

S3 Dataset Excel file containing numerical data for Fig 3. (XLSX)

S4 Dataset Excel file containing numerical data for Fig 4B. (XLSX)

S5 Dataset Excel file containing numerical data for Fig 6A. (XLSX)

S6 Dataset Excel file containing numerical data for Fig 6B. (XLSX)

S7 Dataset Excel file containing numerical data for Fig 7C. (XLSX)

S8 Dataset Excel file containing numerical data for Fig 8F. (XLSX)

S9 Dataset Excel file containing numerical data for Fig 8G. (XLSX)

S10 Dataset Excel file containing numerical data for Fig 9A. (XLSX)

S11 Dataset Excel file containing numerical data for Fig 9B. (XLSX)

S12 Dataset Excel file containing numerical data for Fig 9C. (XLSX)

S13 Dataset Excel file containing numerical data for Fig 9D. (XLSX)

S14 Dataset Excel file containing numerical data for Fig 10B. (XLSX)

S15 Dataset Excel file containing numerical data for S5 Fig. (XLSX)

S16 Dataset Excel file containing numerical data for S6C Fig. (XLSX)

S17 Dataset Excel file containing numerical data for S6D Fig. (XLSX)

S18 Dataset Excel file containing numerical data for S10B Fig. (XLSX)

S19 Dataset Excel file containing numerical data for S10C Fig. (XLSX)

S20 Dataset Excel file containing numerical data for S10D Fig. (XLSX)

S21 Dataset Excel file containing numerical data for S10E Fig. (XLSX)

S22 Dataset Excel file containing numerical data for S12B Fig. (XLSX)

S23 Dataset Excel file containing numerical data for S13A Fig. (XLSX)

S24 Dataset Excel file containing numerical data for S13B Fig. (XLSX)
