# Supplementary figures and images for: Ydj1 interaction at nucleotide-binding-domain of yeast Ssa1 impacts Hsp90 collaboration and client maturation
Source: PLoS Genet. 2022 Nov 9;18(11):e1010442. doi: 10.1371/journal.pgen.1010442 (PMC9645627; doi:10.1371/journal.pgen.1010442)

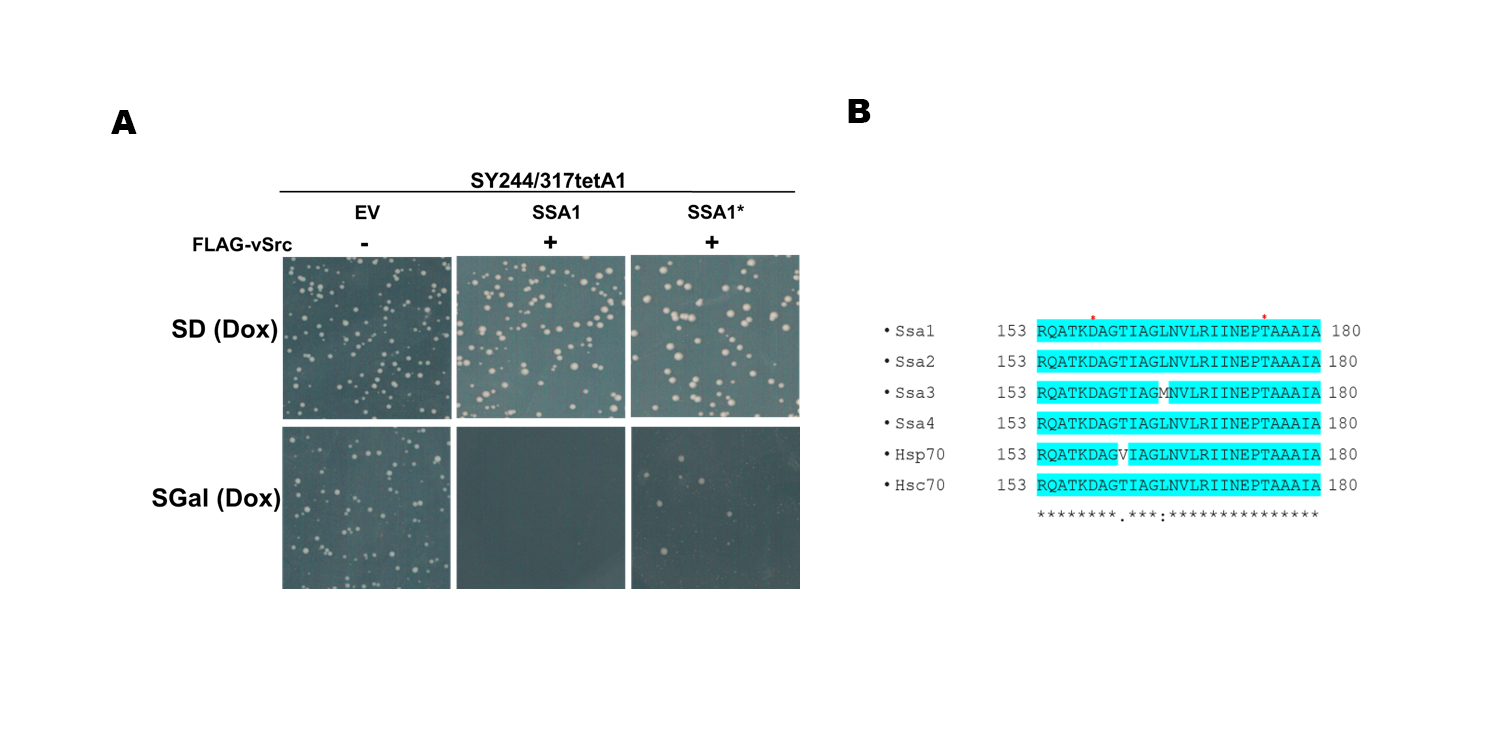

Supplement: S1 Fig — (A) Strain SY244/pRS317-PTetoff-SSA1 was transformed with pRS316 or pRS316-PGAL1-vSrc along with either wt-SSA1 or SSA1 mutagenized with hydroxylamine. Cells were plated onto doxycycline containing SD or SGal plates to shut expression of SSA1 under Tet repressible promoter. Shown is growth after 5 days of incubation at 30°C. (B) Conservation of the residues near identified Hsp70 mutants in Hsp70 isoforms from S. cerevisiae (Ssa1-Ssa4) and Human (Hsp70 and Hsc70). (TIF) [file pgen.1010442.s001.tif]

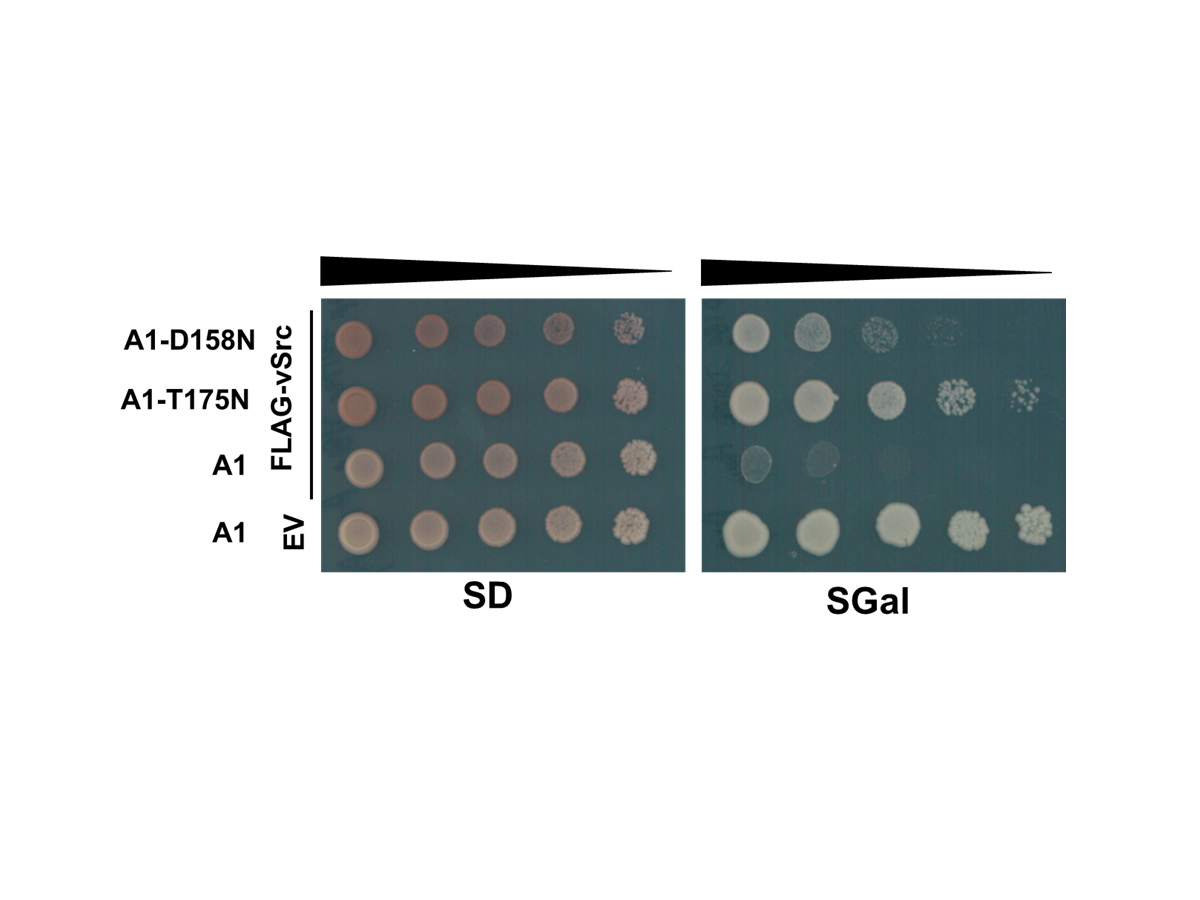

Supplement: S2 Fig — S. cerevisiae strains A1, A1-T175N and A1-D158N were transformed with (EV) or pRS316PGAL1-FLAG-v-Src (FLAG-v-Src). Transformants were pooled and grown into selective SD media. Cells were washed and serially diluted onto SD and SGal media. Shown is growth after 5 days of incubation at 30°C. (TIF) [file pgen.1010442.s002.tif]

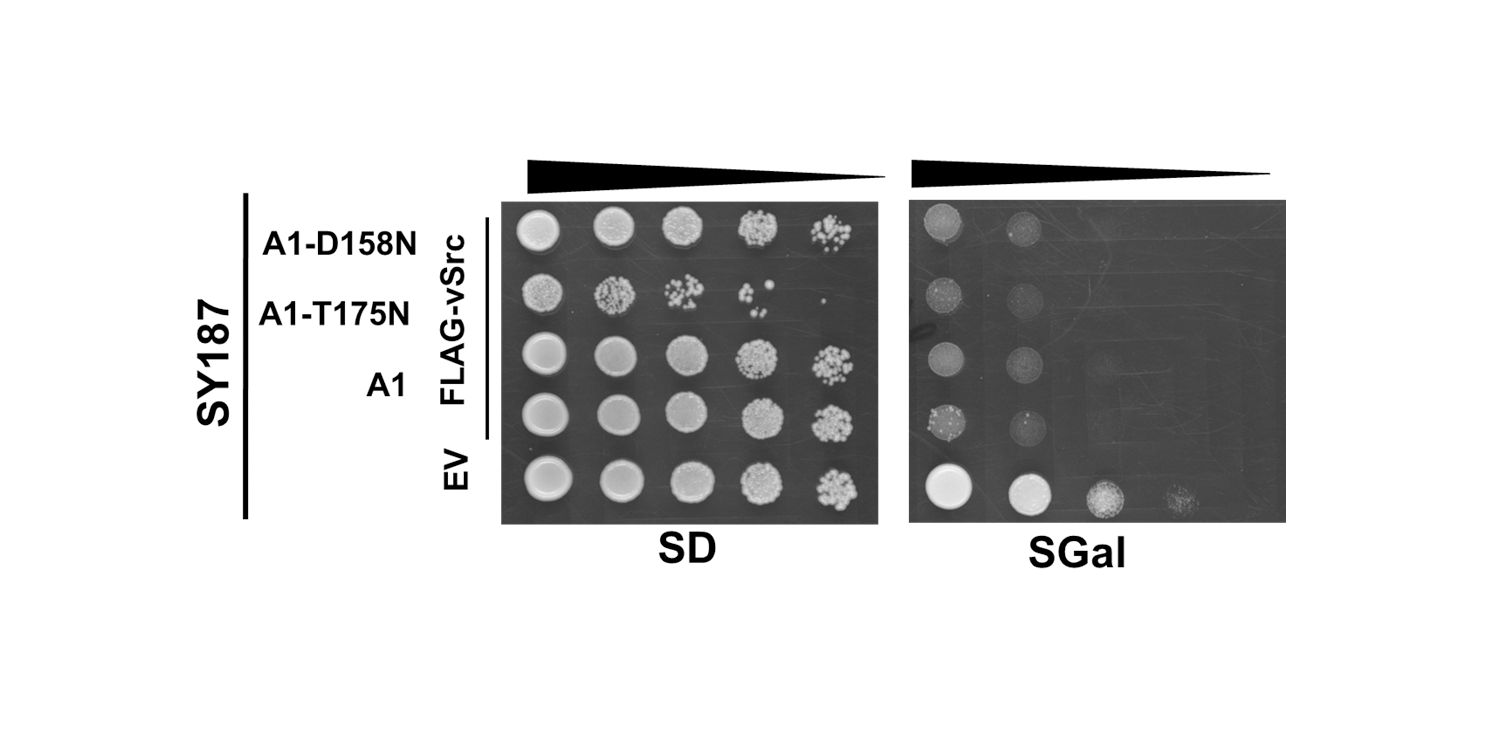

Supplement: S3 Fig — S. cerevisiae strain SY187 was transformed with pRS316 (EV) or pRS316PGAL-FLAG-v-Src (FLAG-v-Src) and pRS315-SSA1 or pRS315-SSA1* (T175N or D158N). Transformants were grown in selective liquid SD media overnight. Cells were further serially diluted and spotted onto SD and SGal media. Shown here is the growth after 4 days of incubation at 30°C. (TIF) [file pgen.1010442.s003.tif]

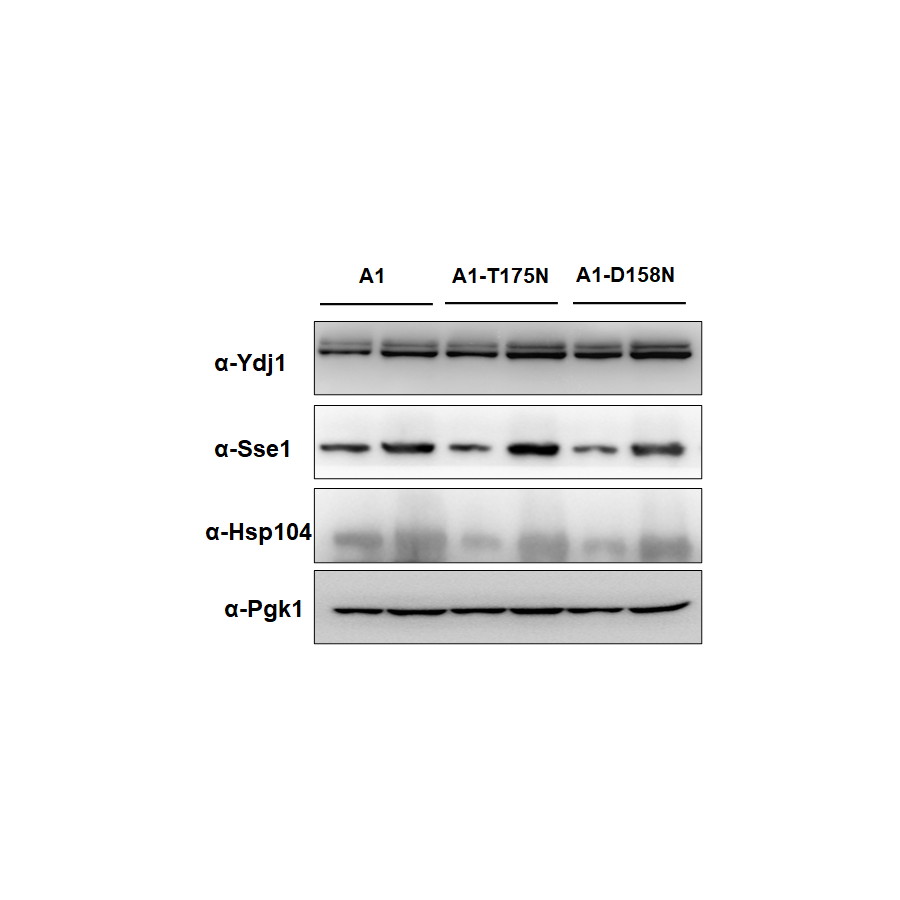

Supplement: S4 Fig — Yeast lysate was prepared from indicated strains grown in inducible liquid SGal media for 6 hours. 5μg (1X) or 10μg (2X) of total lysate protein was loaded into each lane and probed with anti-Ydj1, anti-Sse1, anti-Hsp104, and anti-Pgk1 antibodies. (TIF) [file pgen.1010442.s004.tif]

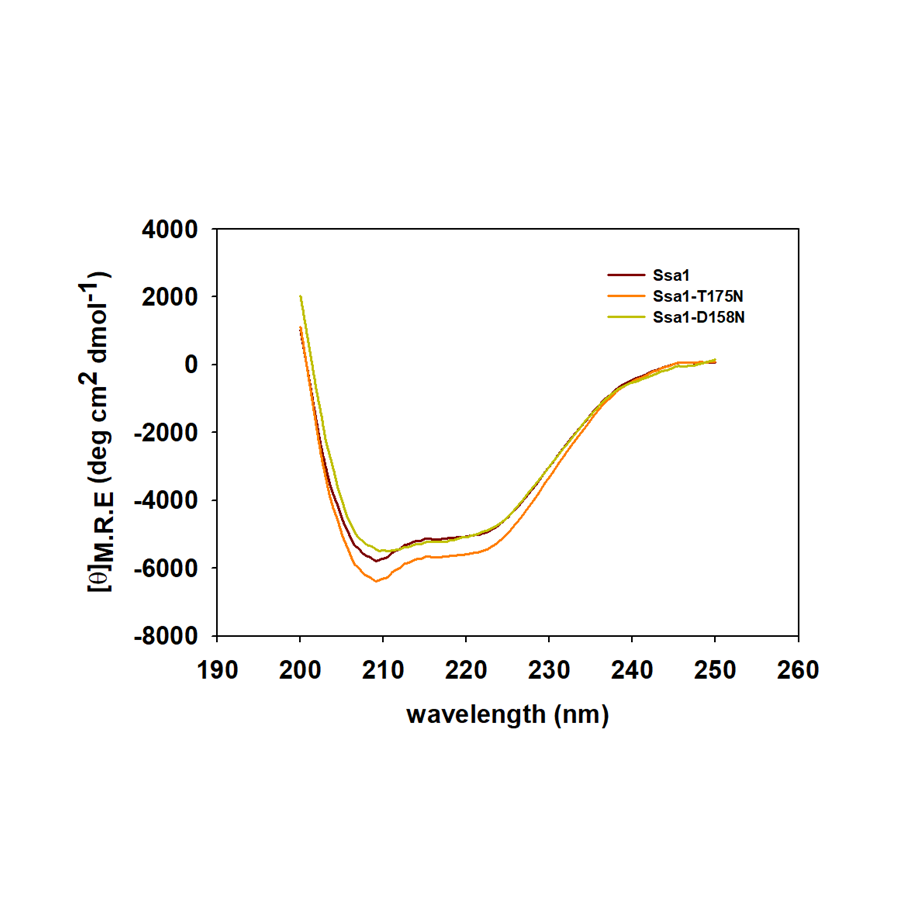

Supplement: S5 Fig — Far-ultraviolet circular dichroism spectroscopic analysis of wt-Ssa1, Ssa1-T175N and Ssa1-D158N. The 10 μM protein in 2.5 mM HEPES buffer containing 15 mM NaCl, pH 7.5 in a 1-mm path length cuvette was used to record CD spectra. (TIF) [file pgen.1010442.s005.tif]

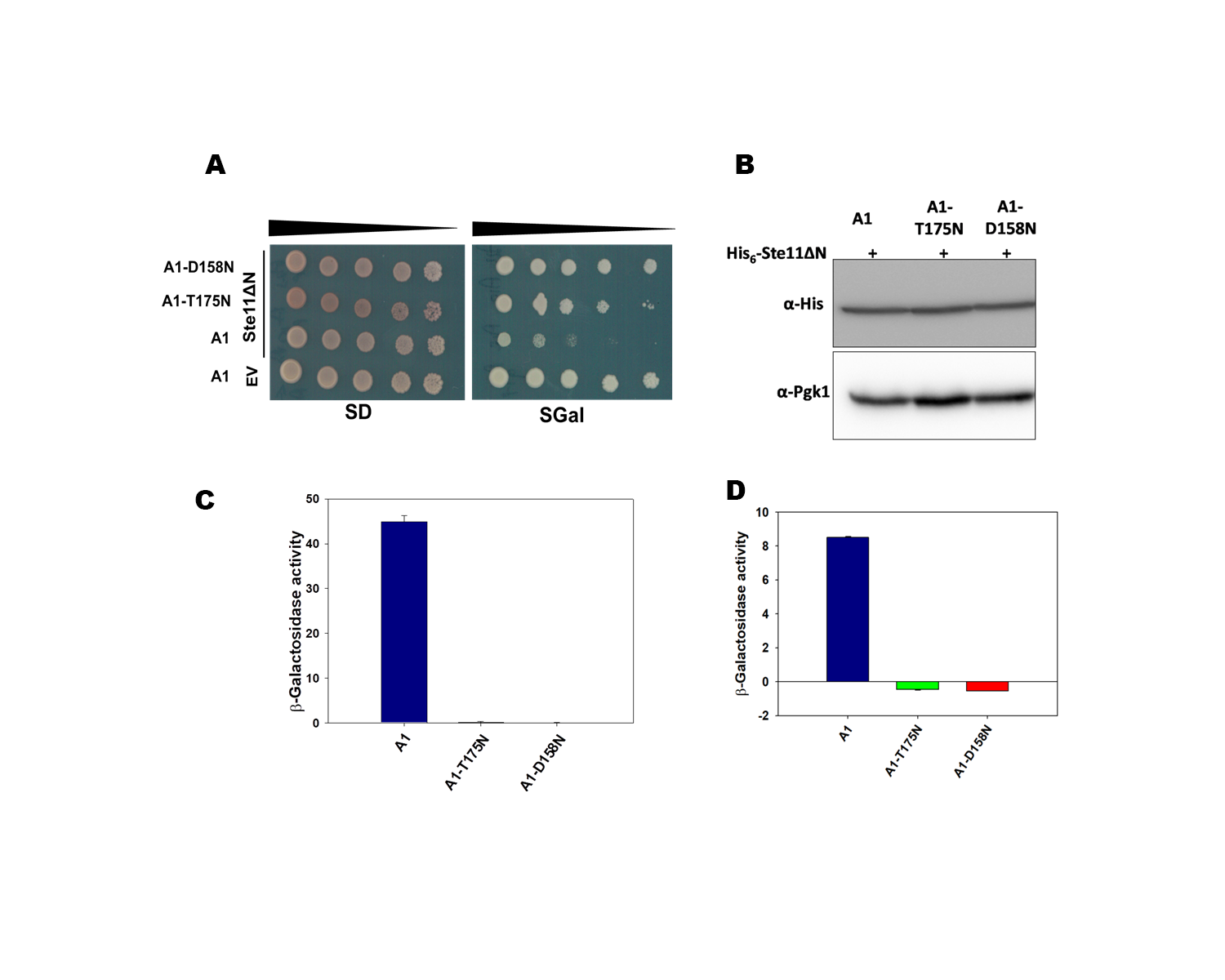

Supplement: S6 Fig — (A) Cells harboring Ste11ΔN encoding plasmid were grown into selective liquid SD media, and further serially diluted onto solid SD and SGal media. Shown is growth after 4 days of incubation at 30°C. (B) Immunoblot showing His6-Ste11ΔN steady-state level in indicated strains. The anti-His6 was used as the primary antibody. (C) Indicated strains were co-transformed with plasmid overexpressing Ste11ΔN and pPRE-lacZ. Ste11ΔN was induced by growing in SGal media. β-galactosidase activity was measured similar to as described above for Ste11. (D) Indicated strains harboring plasmid pPRE-lacZ were grown in SD liquid media. Shown is the β-galactosidase activity measured with similar number of cells harvested during mid-log phase. The activity was calculated in Miller units. Error bars represent standard error from 3 different biological replicates. (TIF) [file pgen.1010442.s006.tif]

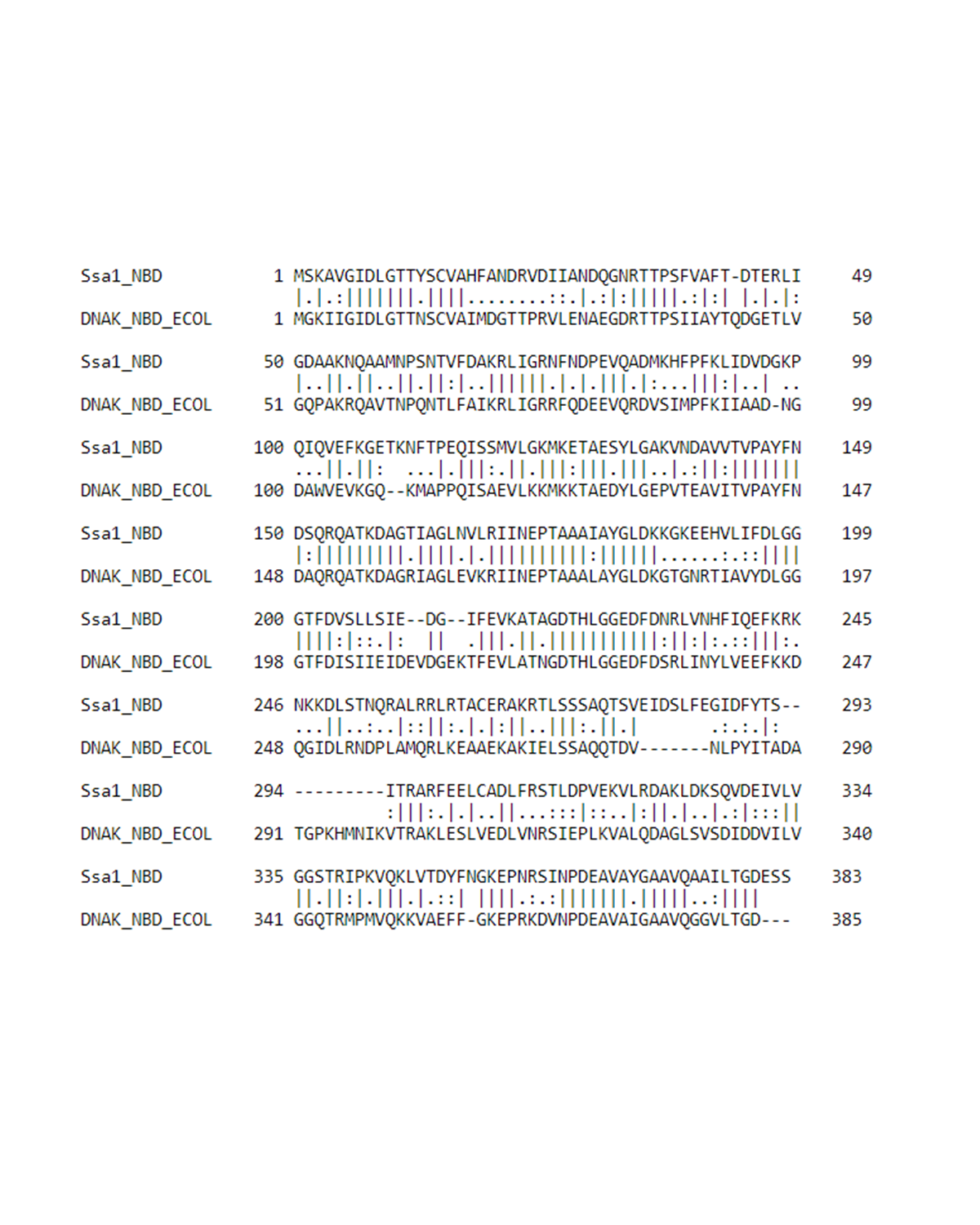

Supplement: S7 Fig — (TIF) [file pgen.1010442.s007.tif]

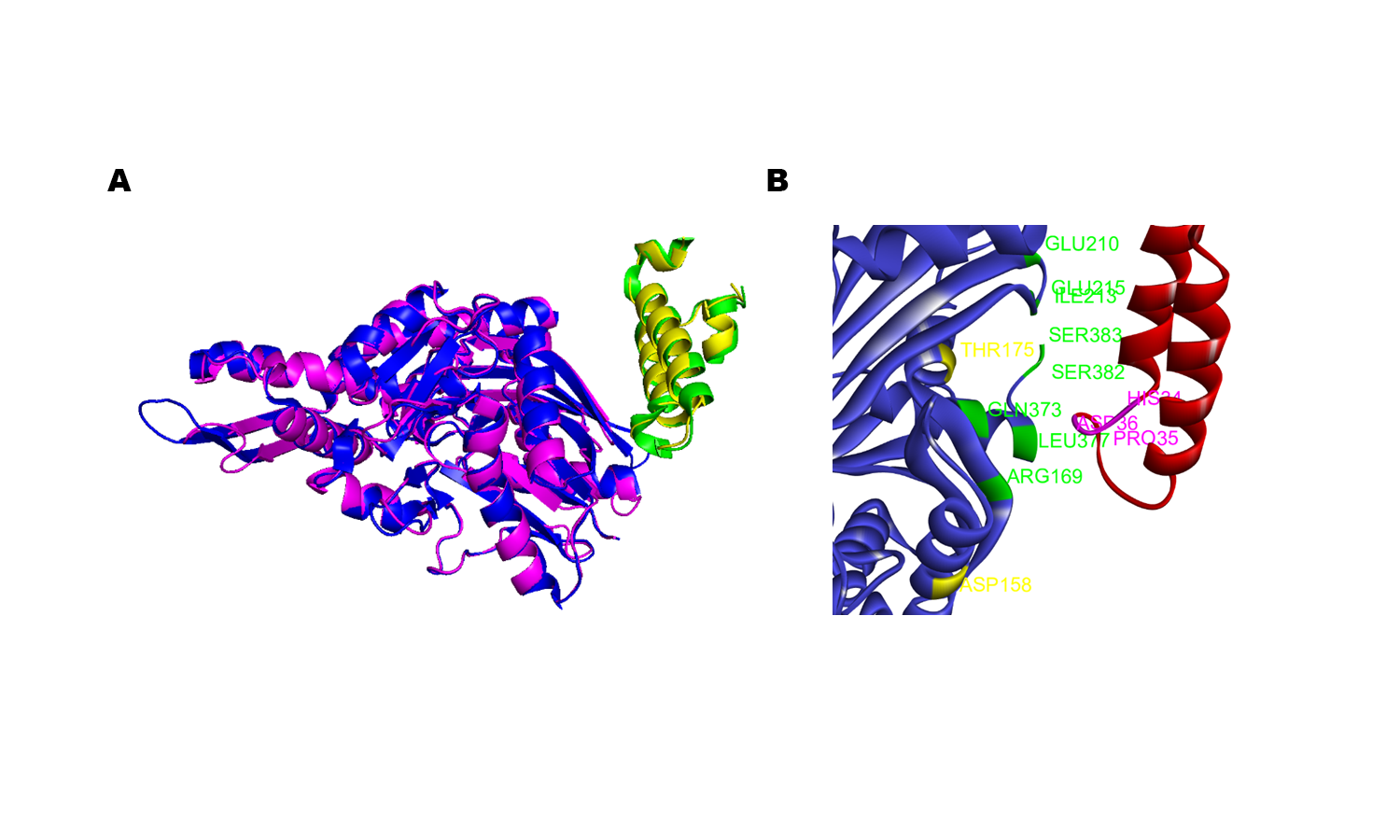

Supplement: S8 Fig — (A) Superposed 3D structures of docked NBD(Hsp70)-J-domain (Hsp40) complex from S.cerevisiae (magenta) and from E.coli (blue). Structure for E.coli Hsp70 and Hsp40 are from PDB ID 5NRO. The RMSD of this alignment is 0.475 Å. (B) the relevant residues are highlighted in modelled complex of S. cerevisiae NBD of Ssa1 (blue) and J domain of Ydj1 (red). Q373, L377, E215, I213, E210 and R169 in green are interacting residues of NBD and H34, P35 and D36 in magenta is HPD motif of J-domain. Residues D158 and T175 are also shown in yellow. (TIF) [file pgen.1010442.s008.tif]

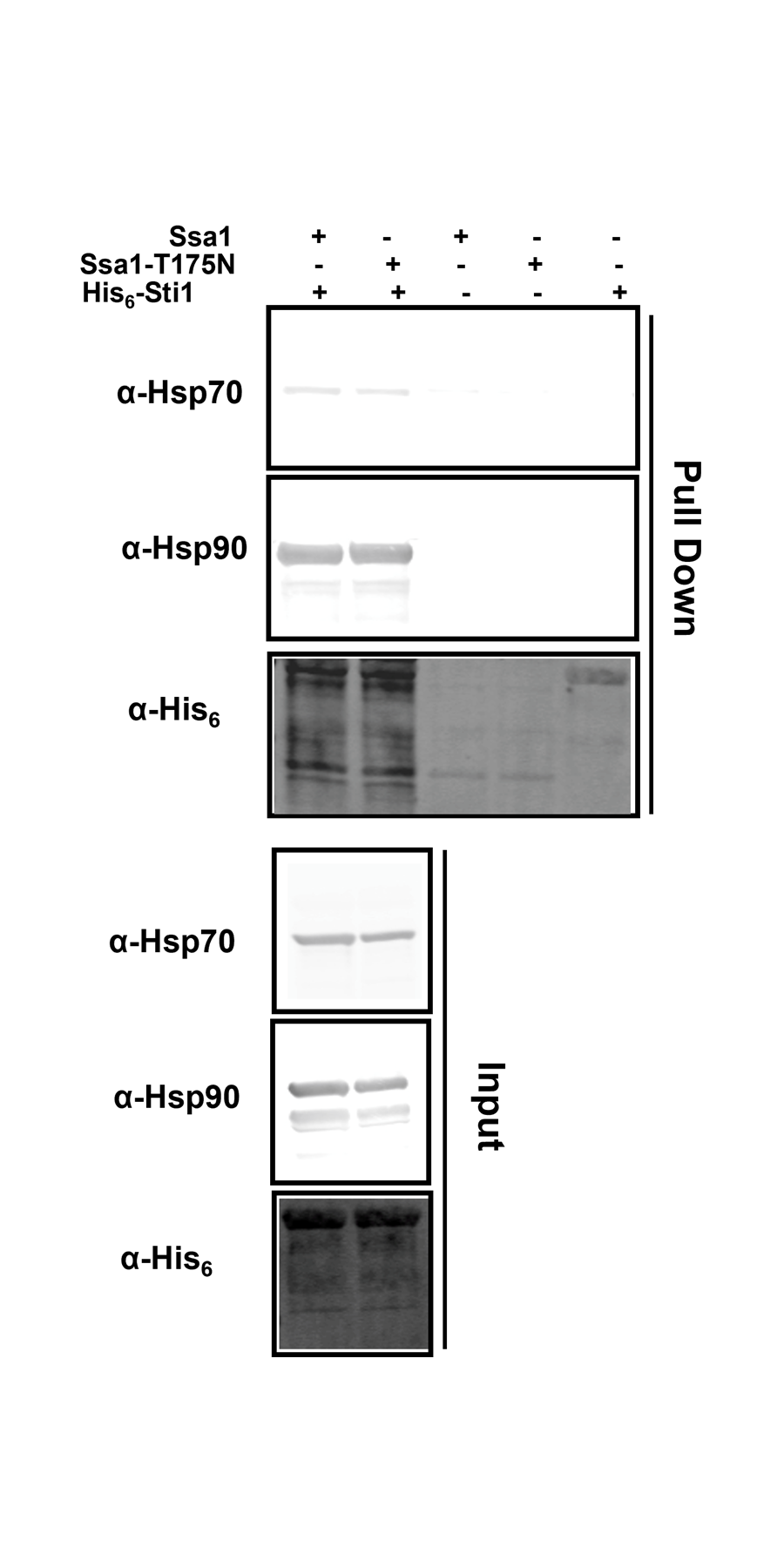

Supplement: S9 Fig — Purified His6-Sti1 was incubated with yeast lysate harbouring Ssa1 and Ssa1 T175N as sole source of cytosolic Hsp70. Bound His6-Sti1 was further incubated with Co2+-NTA resin. Precipitated proteins were immunoblotted with antibodies against Hsp70, Hsp90 and His6. (TIF) [file pgen.1010442.s009.tif]

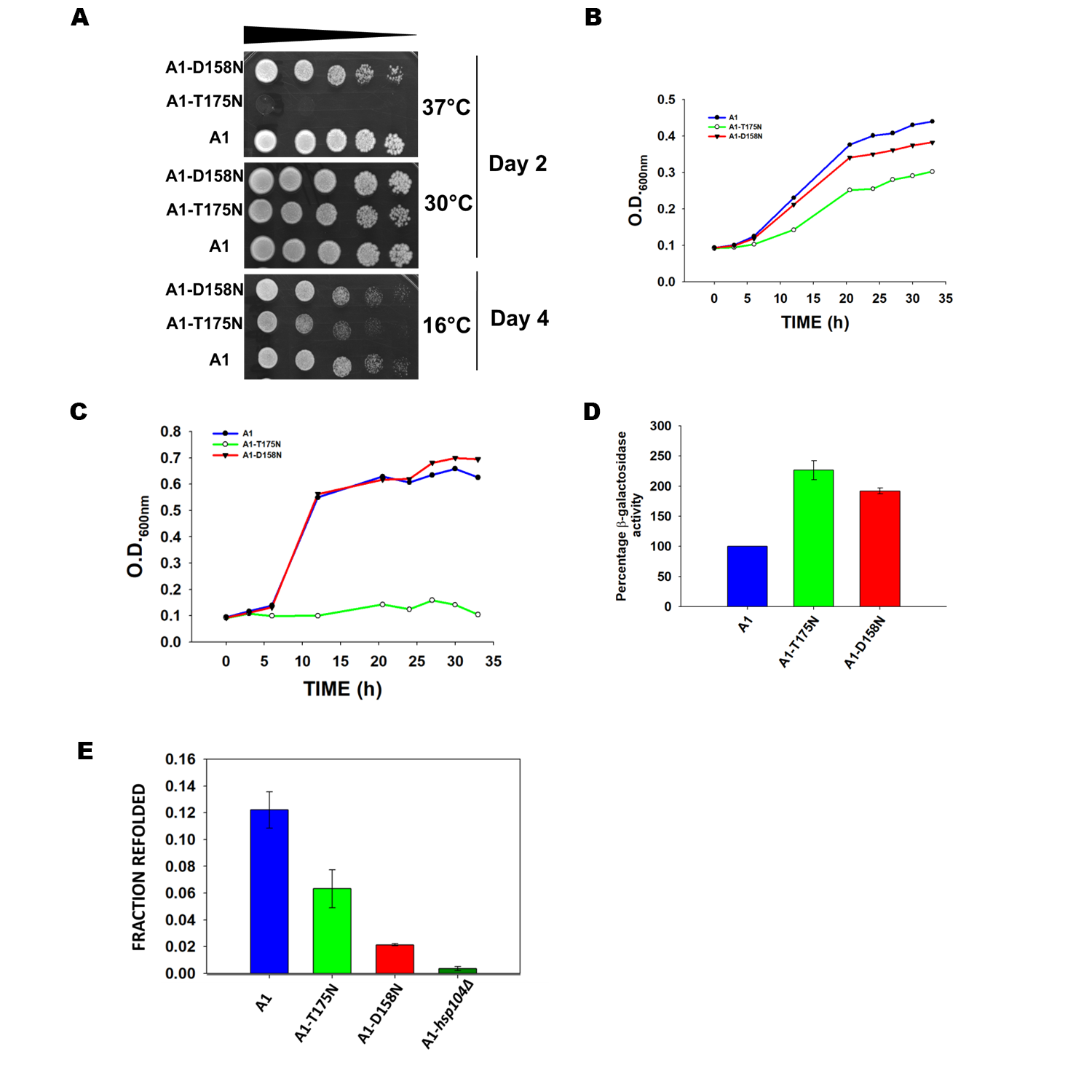

Supplement: S10 Fig — (A) A1, A1-T175N or A1-D158N strains were serially diluted and spotted onto YPAD plates. The plates were incubated at 16°C for 5 days, 30°C, and 37°C for 2 days. (B and C) S. cerevisiae strains were grown at 30°C and 37°C respectively. Shown here is O.D.600nm at indicated time points. (D) The indicated S. cerevisiae strains harboring plasmid encoding HSE-lacZ was grown in liquid growth media at 30°C. Subsequently, cells were shifted for heat shock at 37°C for 4 hours. Cells count was normalized and further assayed for β-galactosidase activity as described in material and methods. (E) S. cerevisiae strains expressing thermolabile firefly luciferase were grown at 30°C. Cells were shifted to 48°C for 30min. The refolding was initiated by further incubating cells at 30°C. Shown is the fraction refolded at 30°C in 60 min as compared to that before shifting cells to 48°C. Error bars represent standard error from 3 different biological replicates. (TIF) [file pgen.1010442.s010.tif]

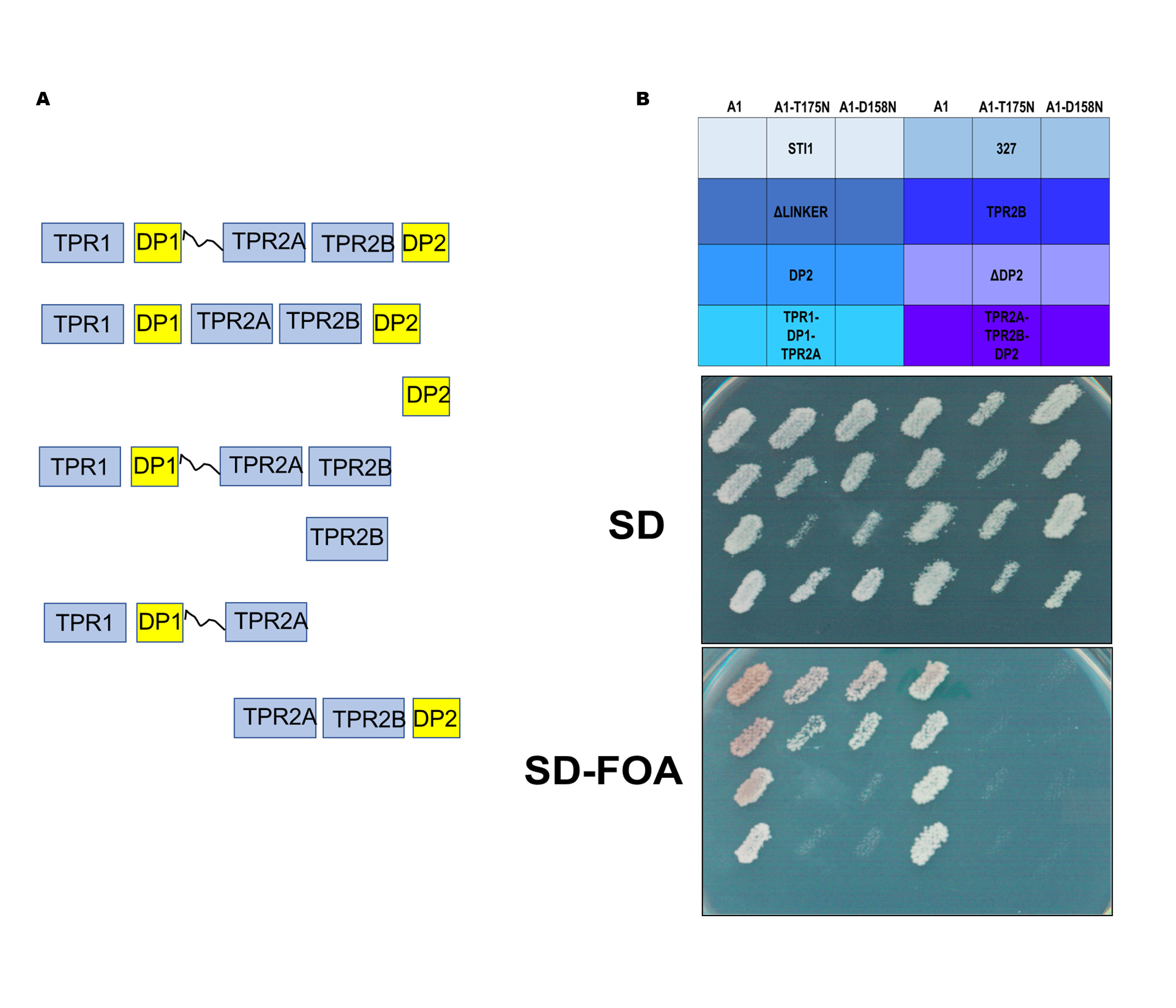

Supplement: S11 Fig — (A) Schematic of domain arrangement of Sti1 and its variants used in the study. (B) The sti1Δ strains expressing Ssa2 (on Ura3 based plasmid), Ssa1 or its variants (on Leu2 based plasmid), and Sti1 (on Lys2 based plasmid) or its variants were grown onto SD plates and further patched onto FOA plates. The individual Sti1 variant is represented by the same color-code. (TIF) [file pgen.1010442.s011.tif]

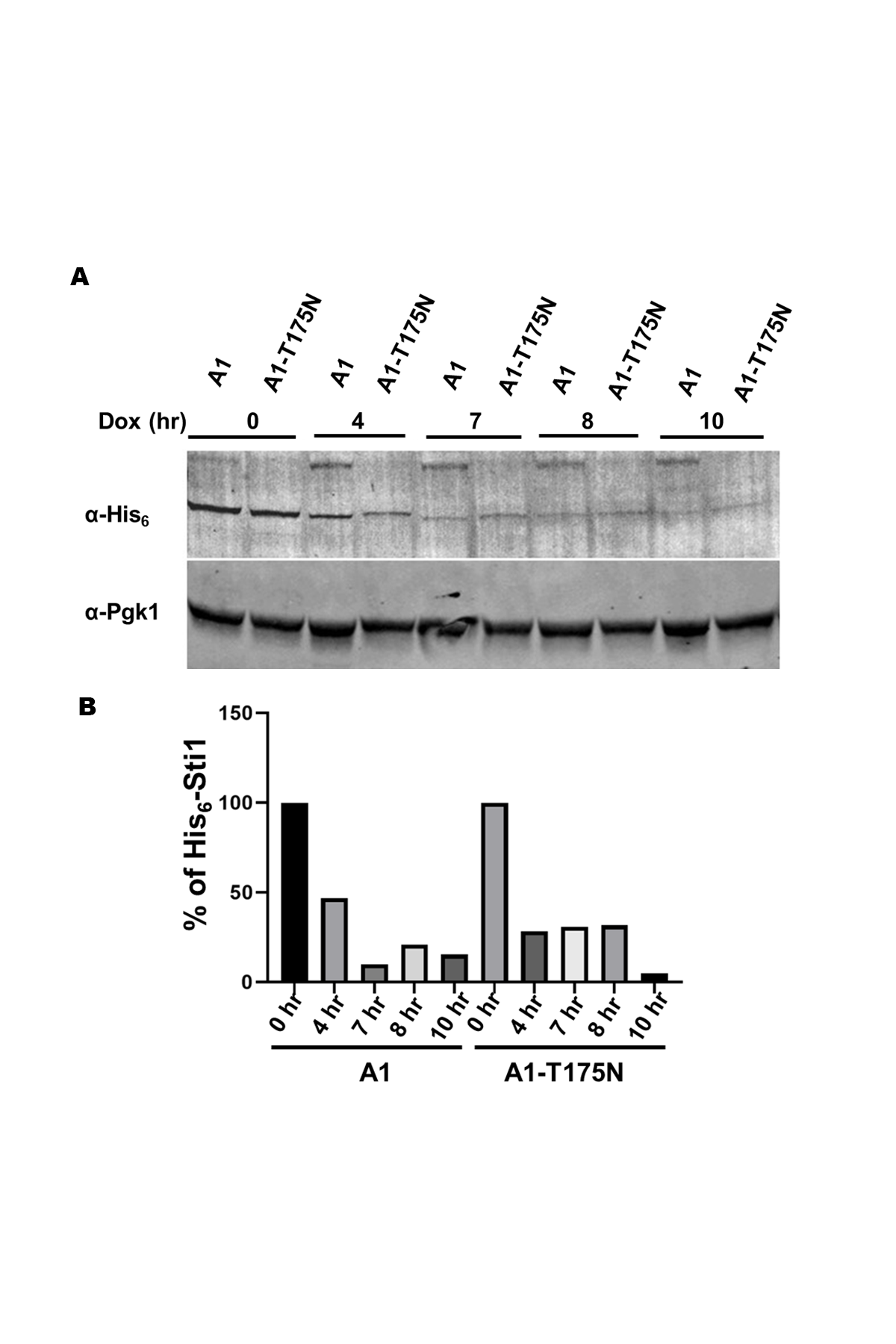

Supplement: S12 Fig — (A) His6-Sti1 was expressed from TET repressible promoter in A1 and A1-T175N strains. The doxycycline (repressor) was added in the growth media and His6-Sti1 abundance was monitored at indicated time intervals. (B) Graph showing the percentage repression of His6-Sti1 at different time points with respect to 0 hr in A1 and A1-T175N strains. (TIF) [file pgen.1010442.s012.tif]

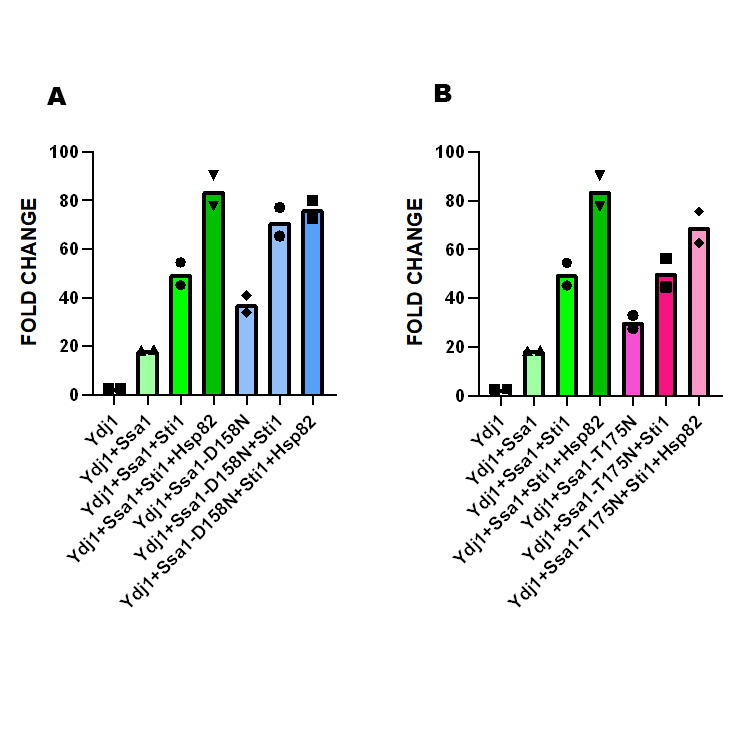

Supplement: S13 Fig — (A) and (B) The bar graph representation of the study shown in Fig 9A and 9B respectively at 30min. Briefly, the denatured luciferase (40nM) was incubated in the presence of 0.3μM Ydj1, 0.5μM Hsp70 (Ssa1, Ssa1-T175N or Ssa1-D158N), 2.5μM Sti1 and 0.9μM Hsp82. Shown here is fold change in luminescence with respect to denatured luciferase after 30 minutes. The bar graph shows the average of the 2 replicates along with the data points. Controls are same as for S13A and S13B Fig. (TIF) [file pgen.1010442.s013.tif]
